# Recombinant ROP8 antigen: diagnostics, immunogenicity, and therapeutic targeting in toxoplasmosis

**DOI:** 10.3389/fimmu.2026.1768856

**Published:** 2026-02-05

**Authors:** Maciej Chyb, Adrian Bekier, Malwina Kawka, Bartłomiej Ferra, Bożena Dziadek, Sylwia Michlewska, Katarzyna Dzitko, Nazar Trotsko, Justyna Gatkowska

**Affiliations:** 1 Department of Molecular Microbiology, Faculty of Biology and Environmental Protection, University of Lodz, Lodz, Poland; 2 The Bio-Med-Chem Doctoral School of the University of Lodz and Lodz Institutes of the Polish Academy of Sciences, Faculty of Biology and Environmental Protection, University of Lodz, Lodz, Poland; 3 Department of Microbiology and Laboratory Medical Immunology, Medical University of Lodz, Lodz, Poland; 4 Department of Tropical Parasitology, Institute of Maritime and Tropical Medicine in Gdynia, Medical University of Gdańsk, Gdynia, Poland; 5 Institute of Molecular Biology and Biotechnology (IMBB), Foundation for Research and Technology – Hellas (FORTH), Heraklion, Greece; 6 Laboratory of Microscopic Imaging and Specialized Biological Techniques, Faculty of Biology and Environmental Protection, University of Lodz, Lodz, Poland; 7 Department of Organic Chemistry, Faculty of Pharmacy, Medical University of Lublin, Lublin, Poland

**Keywords:** innate response, ROP8, serodiagnosis, *T. gondii*, thiazolidin-4-one derivatives

## Abstract

**Introduction:**

*Toxoplasma gondii* invasion relies on the battery of specialized proteins, such as ROP8, which plays a key role in formation of parasitophorous vacuoles within host cells. The aim of the work was to evaluate diagnostic, immunoprophylactic and therapeutic potential of recombinant ROP8 (rROP8) protein as despite many years of research this parasitosis still is considered a significant medical and economic problem and the main areas requiring improvement are the diagnostics of the infection, vaccine development especially for humans and safe, specific chemotherapy.

**Methods:**

Recombinant ROP8 protein (rROP8) of *Toxoplasma gondii* was cloned, expressed in *E. coli* Rosetta(DE3)pLysS, and purified by nickel-affinity chromatography. Its diagnostic potential was evaluated using ELISA on sera from experimentally infected mice and human patients. The immunogenic properties of rROP8 were assessed through *in vitro* stimulation of human monocytes, mouse macrophages, and monocyte-derived dendritic cells, followed by cytokine profiling and co-culture with autologous CD4^+^ lymphocytes. Finally, drug affinity responsive target stability (DARTS) assays and confocal microscopy were used to investigate the interaction of thiazolidin-4-one derivatives with rROP8 and their inhibitory effects on parasitophorous vacuole formation.

**Results:**

The ROP8 proved to be promising as a potential diagnostic tool enabling the detection of specific IgG antibodies in mouse and human immune sera comparably to the native parasite antigen (TLA). The protein was also capable of activating human monocytes and mouse macrophages *in vitro* leading to the enhanced clearance of *T. gondii* tachyzoites via phagocytosis. The protein was also able to activate human monocyte-derived dendritic cells as shown by the cytometric analysis of surface markers and multiplex detection of specific cytokines, also in co-cultures with homologous lymphocytes, leading to Th1/Th17 lymphocyte phenotype. Finally, the proteolytic degradation of rROP8 was significantly inhibited by the presence of all thiazolidin-4-one derivatives, which exhibited high activity against *T. gondii* in previous studies.

## 1 Introduction

Approximately 30% of the human population is infected by apicomplexan, food-born parasite *Toxoplasma gondii*, which is an etiological factor of toxoplasmosis. Human prevalence varies between countries and regions, from 10% to as much as 80%, depending on climatic factors, socioeconomic status, or hygiene conditions, and it is directly linked to routes of *T. gondii* transmission (1). Humans can become infected in several ways: by eating raw or undercooked meat containing tissue cysts, by ingesting oocysts in contaminated water or on vegetables and fruits, and through blood transfusions or organ transplants (2). Other important routes include vertical infection of the fetus, which can occur when tachyzoites cross the placenta. Globally, the transmission rate is estimated at 29% and the stage of pregnancy is important for the development of clinical symptoms. The parasite can cross the placenta more easily in the last trimester, on the other hand there is less chance of severe symptoms occurring and these include hydrocephalus or microcephalus, intracranial calcification and retinochoroiditis. In the early weeks of gestation, congenital toxoplasmosis can lead to spontaneous abortion (3). In general, about 80% of immunocompetent people are asymptomatic. Immunocompromised individuals may develop non-specific clinical signs such as fever, cervical lymphadenopathy, myalgia, and less commonly, toxoplasmic retinochoroiditis due to acquired toxoplasmosis (about 23% of all cases of ocular toxoplasmosis). The severity of infection varies depending on strain genotype, however for immunocompromised patients, toxoplasmosis is a life-threatening disease, and that group suffers mostly from disease reactivation (1). Due to the fact that the parasite is present in the central nervous system, since 1950 many studies were performed to link this infection with neuropsychiatric diseases including schizophrenia, Alzheimer`s disease, bipolar disorder, epilepsy, and obsessive-compulsive disorder. While studies and meta-analyses agree on the link with schizophrenia, other disorders are still questionable (4). However, the potential impact on behavioral changes and systemic disease cannot be underestimated. Toxoplasmosis also has a direct economic impact on livestock farming, especially sheep, goats and pigs, by causing abortions or weight loss in infected animals (5). The parasite has a multi-stage, sexual and asexual complex life cycle. We can distinguish three infectious stages, bradyzoites in tissue cysts, sporozoites in oocysts, and tachyzoites present in blood during acute infection. These forms differ in morphology, speed of multiplication which translates into differences in gene expression profiles between each stage (6). The protozoan expresses various secretory and non-secretory factors important in cell adhesion and invasion, parasitophorous vacuole and cyst formation, immune response modulation, apoptosis inhibition and autophagy modulation (7).

Rhoptry proteins (ROP) are secretory proteins, released from club-shaped organelles called rhoptries. These factors play a key role in parasite invasion, by taking part in formation and biogenesis of parasitophorous vacuole and are rapidly secreted after microneme proteins synthesis during invasion. ROP2 family is the largest fraction of proteins in rhoptry organelles, and these include ROP2, ROP4, ROP5, ROP7, ROP8 and ROP18 proteins. They take part in many important invasion mechanisms such as linking host cell mitochondria and parasitophorous vacuole, allowing immune evasion by inhibiting accumulation of immunity-related GTPases and downregulating type 1 adaptive immune response and also contributing in the uptake of iron form host cell (8, 9). These proteins contain conserved serine/threonine (S/T) kinase domain, but some members lack kinase activity such as ROP8 (10). ROP8 is 575 amino acid residues type I transmembrane protein with calculated molecular weight of approx. 65.4 kDa, and one of the most abundant proteins from ROP2 family (8, 11). It is considered as a main factor of *T. gondii* acute virulence (11). Expression of this protein is found in both bradyzoites and tachyzoites (12). Bioinformatic analysis performed by Fourtan et al. showed that ROP8 is an immunogenic and non-allergenic protein (11). Due to the abundance of this protein in the parasite, it is being studied for its usefulness in diagnostics and immunoprophylaxis of toxoplasmosis (12–14). *In silico* analysis performed by Abdizadeh et al. showed that thiazolidin-4-one core derivates possess inhibitory properties towards ROP8 protein, suggesting a potential therapeutic target (15). In this study we have taken a multidisciplinary approach to thoroughly assess purified recombinant ROP8 protein (rROP8) for its potential diagnostic usefulness, immunogenic potential *in vitro* and interaction with 4-arylthiosemicarbazide derivatives potent against *T. gondii*.

## 2 Materials and methods

### 2.1 Animals

Sera used in the study were obtained as described previously (16). Briefly, BALB/c inbred male mice 8–12 weeks old were bred under SPF, stable conditions in the Faculty of Biology and Environmental Protection. Sera were isolated from *T. gondii* DX strain infected mice at 0, 2-, 3-, 6- and 12-weeks post infection which corresponded to uninfected (0 weeks) and acute (2 weeks), late acute (3 weeks) or chronic toxoplasmosis phase (6 and 12 weeks).

Experimental procedures carried out on inbred mice were approved by the Polish Local Ethics Commission for Experiments on Animals in Łódź (Agreement 75/ŁB639/2012), which operates under the Law on the Protection of Animals Used for Scientific or Educational Purposes and also conforms to European Directive 2010/63/EU of the European Parliament and of the Council of 22 September 2010 on the protection of animals used for scientific purposes. The manuscript was prepared according to the ARRIVE guidelines 2.0 (17).

### 2.2 Parasites

Three *T. gondii* strains were used in the study: a low-virulence DX strain used for mouse infection, maintained *in vivo* and two variants of highly virulent RH strain maintained *in vitro* on Hs27 human fibroblasts (ATCC- CRL-1634, Manassas, VA, USA) - RH (ATCC-50174, ATCC, Manassas, VA, USA) used as a source of native parasite antigen (TLA) and RH-GFP strain (ATCC-50940, ATCC, Manassas, VA, USA) used to determine macrophage phagocytic activity *in vitro.* The TLA was prepared as described previously (16) from repeatedly frozen and thawed RH strain tachyzoites.

### 2.3 *T. gondii* ROP8 recombinant protein preparation

Gene fragment of interest were amplified using Phusion High-Fidelity DNA Polymerase (Thermo Fisher Scientific, Waltham, MA, USA) from cDNA obtained by first strand reverse transcription reaction using a commercially available kit (A&A Biotechnology, Gdynia, Poland). The RNA template was isolated from the RH strain using a commercially available kit (A&A Biotechnology, Gdynia, Poland). The final PCR product was obtained using a standard PCR amplification protocol with Phusion High-Fidelity DNA Polymerase (Thermo Fisher Scientific, Inc., Waltham, MA, USA) using designed oligonucleotide primers forward 5′-TGGACAGCCCAGATCGCCACGTACAGCAAGGCG-3′ and reverse 5′-ATCGGTACCCAGATCTGCCGGTTCTCCA-3′ (Genomed, Warsaw, Poland). The amplicon was inserted into the pET30 Ek/LIC vector, *Bgl*II site (36–575 aa *KM973070.1*). The full sequence of the resulting protein is included in the **Supplementary Figure 4**. Cloning was performed using the In-Fusion HD Cloning Kit (Takara Bio Inc., Kasatsu, Shiga, Japan).

Recombinant proteins were produced using the *E. coli* strain Rosetta(DE3)pLysS. Production was carried out at 30 °C for 16 hours after IPTG induction. Proteins were purified on a metal affinity Ni-Sepharose™ 6 Fast Flow column (Cytiva, Little Chalfont, England, United Kingdom) according to the manufacturer’s protocol. Cell pellets from 100 ml culture were resuspended in 30 ml buffer (5 M urea, 20 mM Tris, 0.5 M NaCl, 5 mM imidazole, 0.1% Triton X-100) and sonicated. After centrifugation, the protein extracts obtained by dissolving the inclusion bodies with urea were transferred to Ni columns, washed with buffers containing increasing concentrations of imidazole, and eluted with buffer without urea (20 mM Tris, 0.5 M NaCl, 0.5 M imidazole, 0.1% Triton X-100 pH 7.9). The purification resulted in electrophoretically homogeneous protein preparations (**Supplementary Figure 5**), with a purity of over 90% based on SDS-PAGE. Molecular weight (70.58 kDa) and PI (7.04) were calculated using the website https://web.expasy.org/compute_pi/. Protein concentration was determined using commercially available Bradford reagent (Merck KGaA, Darmstadt, Germany).

For cell culture studies, Pierce High Capacity Endotoxin Removal Spin Columns were used to remove potential LPS contamination using overnight manufacturer’s protocol, along with Pierce Chromogenic Endotoxin Quant Kit (Thermo Fisher Scientific, Waltham, MA, USA).

### 2.4 Human sera

All serum samples were obtained from the collection of the Department of Tropical Parasitology of the Medical University of Gdańsk, Poland on completely anonymized samples of biological material collected from patients of the University Centre for Maritime and Tropical Medicine as part of routine laboratory diagnostics, from whom consent for use for scientific purposes was obtained. All procedures performed in this study were in accordance with the ethical standards of the institutional research committee and the 1964 Helsinki Declaration and its later amendments or comparable ethical standards. Serum samples were defined as *T. gondii* seropositive or seronegative based on commercial test results (VIDAS TOXO IgM, VIDAS TOXO IgG II and VIDAS TOXO IgG AVIDITY bioMérieux, Marcy l’Etoile, France). Seronegative sera (n=138) constituted a control group, and seropositive sera were further divided into acute (n=50) and chronic groups (>200 IU/ml n=40, 101–200 IU/ml n=27, <100 IU/ml n=77).

### 2.5 Reactivity of immune mouse and human sera

As described previously (16, 18) the potential diagnostic utility of rROP8 protein to detect specific anti-*T. gondii* antibodies present in mouse and human immune sera were tested with an indirect ELISA test. For the assays, the MaxiSorp plates (Thermo Fisher Scientific, Inc., Waltham, MA, USA) were coated overnight with rROP8 protein at a final concentration of 2.5 µg/ml or TLA at 1 µg/ml. The immune sera were tested at 1:100 dilution and secondary whole IgG anti-mouse IgG and IgM horseradish peroxidase (HRP)-conjugated antibodies (Jackson ImmunoResearch Europe Ltd., Cambridgeshire, UK) or whole IgG anti-human IgG horseradish peroxidase (HRP)-conjugated antibodies (Jackson ImmunoResearch Europe Ltd., Cambridgeshire, UK) were used for mouse and human assay, respectively. Colour reactions were developed with o-phenylenediamine dihydrochloride chromogenic substrate (Merck, KGaA, Darmstadt, Germany) or H_2_O_2_ (Merck, KGaA, Darmstadt, Germany) and ABTS (Merck, KGaA, Darmstadt, Germany) as a chromogene for human and mouse test, respectively. The mean of two technical respects for each serum sample OD was calculated, and the positive reaction was defined as mean OD exceeding the cut-off value defined as men OD of control sera + 2*SD.

The human and mouse sera were examined using an ELISA test to determine their reactivity with lysate from an uninduced *E. coli* Rosetta(DE3)pLysS strain that underwent the same purification steps as the induced strain carrying the plasmid encoding the recombinant protein being tested. The *E. coli* lysate was used at the dilution of the lowest diluted protein preparation. Neither the human nor the mouse sera showed reactivity with the control *E. coli* lysate, and the calculated cut-off values were lower than those obtained for the recombinant protein.

### 2.6 *In vitro* monocyte and macrophages activation

Two cell lines were used: human monocytes bearing the NF-κB-inducible SEAP reporter construct of THP1-Blue cell line (InvivoGen, San Diego, USA) and mouse macrophage cell line ANA1 (CVCL_0142, collection of the Department of Molecular Microbiology, Institute of Microbiology, Biotechnology and Immunology, Faculty of Biology and Environmental Protection, University of Lodz).

The test on THP1-Blue cells was carried out as described previously (19). The cells were maintained at 37 °C in a 5% CO_2_ atmosphere in RPMI 1640 medium (Gibco, Thermo Fisher Scientific, Waltham, MA, USA) supplemented with 2 mM L-glutamine, 25 mM HEPES, 10% heat-inactivated fetal bovine serum (Biowest, Cytogen, Zgierz, Poland), 100 μg/ml normocin (InvivoGen, San Diego, USA) and penicillin-streptomycin (100 U/ml - 100 μg/ml) (Merck KGaA, Darmstadt, Germany). Blasticidin at a concentration of 10 μg/ml (InvivoGen, San Diego, USA) was used as a repressor while the test was performed in a medium without the repressor. LPS from *E. coli* O55:B5 (Merck KGaA, Darmstadt, Germany) at a concentration of 2.5 ng/ml and culture medium were used as positive and negative controls, respectively. Supernatants of rROP8 stimulated cells were incubated with QUANTI-Blue reagent for 6 hours at 37 °C and OD was measured at 650 nm on SpectraMax i3 (Molecular Devices, San Jose, USA).

ANA1 mouse macrophages were grown at 37 °C in a 5% CO2 atmosphere in DMEM medium (Gibco, Thermo Fisher Scientific, Waltham, MA, USA) supplemented with GlutaMAX (Gibco, Thermo Fisher Scientific, Waltham, MA, USA), 10% heat-inactivated fetal bovine serum (Biowest, Cytogen, Zgierz, Poland), penicillin-streptomycin (100 U/ml - 100 μg/ml) (Merck KGaA, Darmstadt, Germany), and plasmocin 5 μg/ml (InvivoGen, San Diego, USA). For the test cells were cultured for 24 h in a 96-well plate at a density of 1x10^5^ cells/well in medium without plasmocin and then stimulated with rROP8 (10 µg/ml), TLA (10 µg/ml) or *E. coli* O55:B5 LPS (10 ng/ml) while cells in culture medium alone constituted a negative control. Supernatants were collected 24 h later for cytokine evaluation. Stimulated macrophages were infected with 1x10^5^ per well RH-GFP tachyzoites, MOI 1:1. This assay was carried out in DMEM medium without phenol red (Gibco, Thermo Fisher Scientific, Waltham, MA, USA) supplemented with 2 mM L-glutamine, 3% double heat-inactivated FBS (Biowest, Cytogen, Zgierz, Poland), penicillin-streptomycin (100 U/ml - 100 μg/ml) (Merck KGaA, Darmstadt, Germany). The *T. gondii* invasion was maintained for 72 h and the percentage of viable tachyzoites was determined based on fluorescence reading on SpectraMax i3 (Molecular Devices, San Jose, USA) at 488/510 nm.

Concentrations of TNF-α (Invitrogen, Thermo Fisher Scientific, Waltham, MA, USA), IL-12p40 (Invitrogen, Thermo Fisher Scientific, Waltham, MA, USA), and IL-10 (OptEIA, BD Biosciences, San Jose, CA, USA) in cell culture supernatants were evaluated with commercially available ELISA sets according to manufacturer’s protocols using non-linear regression.

Cell viability was assessed after each stimulation experiment using the resazurin reduction assay to exclude false negatives due to potential antigen toxicity (**Supplementary Figure 1A**). To exclude false positive results due to contamination of antigen preparations by *E. coli* components, an *E. coli* Rosetta(DE3)pLysS culture was transformed with empty pET30 EK/LIC, induced and purified on a metal affinity column following the standard protocol for antigen purification (**Supplementary Figure 1B**).

For the tests at least 2 separate rROP8 protein batches were used with 4–8 technical repeats in each experiment. Monocyte activation results constitute mean values from 6 independent experiments, macrophage cytokine production mean values from 3 independent experiments, and *T. gondii* viability in stimulated macrophage culture as the mean of 8 independent experiments.

### 2.7 Human dendritic cells assays

#### 2.7.1 Preparation of human monocyte-derived dendritic cells

**Figure 1:**
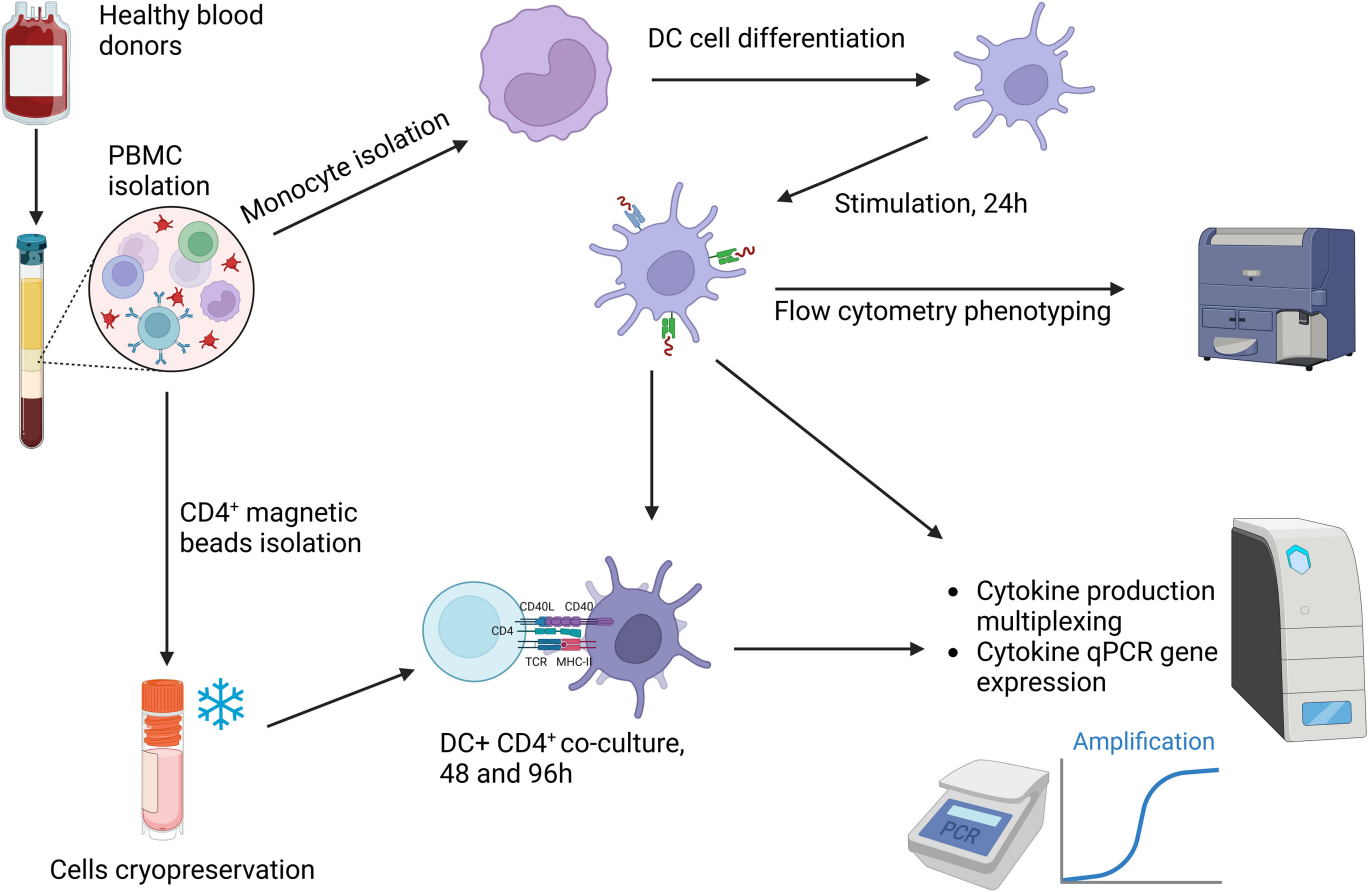
Human dendritic cells and CD4^+^ lymphocytes activation experimental setup. Created in BioRender. Chyb, M. (2026) https://BioRender.com/s14v653.

To differentiate DCs first human monocytes were isolated from anonymized commercially available (Regional Blood Donation Station, Lodz, Poland) buffy coats obtained from 3 healthy human blood donors. A diagrammatic representation of the experimental setup is provided in **Figure 1**. Monocytes were isolated by a double density gradient protocol with Histopaque-1077 (Merck KGaA, Darmstadt, Germany) and 46% iso-osmotic Percoll (Amersham Pharmacia Biotech), as described previously (20). The remaining stages of the experiment were conducted in accordance with the previously established protocol, as described by Krawczyk et al. (21). Briefly, first, the peripheral blood mononuclear cells (PBMC) were isolated from buffy coats on Histopaque-1077 and counted using a trypan blue exclusion test, next human monocytes were obtained from live PBMC layered on an iso-osmotic gradient at a density of 5×10^6^/ml cells. In the meantime, the CD4^+^ T lymphocytes were isolated from PBMC using CytoSinctTM gL Columns (GenScript) and CytoSinctTM CD4 Nanobeads for human cells (GenScript), according to manufacturer’s protocols. Again the number of live monocytes was determined and they were suspended in the IMDM medium (Biowest, Cytogen, Zgierz, Poland) supplemented with 10% Heat-inactivated fetal bovine serum (Biowest, Cytogen, Zgierz, Poland), 100 U/ml penicillin and 100 µg/ml streptomycin (Merck KGaA, Darmstadt, Germany), seeded in the 6-well plates (Falcon) at a density of 1×10^6^ cells/ml and incubated for 6 days with at 37 °C, 10% CO_2_ in the presence of 10 ng/ml IL-4 (R & D Systems) and 25 ng/ml GM-CSF (R & D Systems) to promote their differentiation into DCs. After culture only live, differentiated cells remained in the cultures as verified by inverted light microscopy. Differentiated cells were detached from surface using cell scrapers (Falcon) and ice-cold PBS/2mM EDTA (Merck KGaA, Darmstadt, Germany) and next the cells were used in stimulation assays in duplicate for each donor. Furthermore the 2 ml plasma samples were obtained from centrifuged buffy coats and stored at -20 °C until evaluated with Toxo-Screen DA (bioMérieux, Marcy l’Etoile, France) for the presence of specific anti-*T. gondii* antibodies at 1:40 and 1:4000 dilution. The result of the anti-*T. gondii* screening on blood from donors is presented in the **Supplementary Materials**, **Supplementary Figure 6**.

#### 2.7.2 DC stimulation

Obtained immature DCs were seeded in 12-well plate at the same density of 1×10^6^ cells/ml for each donor and stimulated for 24 hours with recombinant rROP8 protein at the concentration of 10 µg/ml and LPS from *E. coli* O55:B5 at a concentration of 0,5 μg/ml (Merck KGaA, Darmstadt, Germany), which served as a positive control. Non-stimulated cultures, grown in the presence of medium alone were used to determine the background reactivity of DCs. Again, a resazurin reduction assay was performed to exclude a potential toxic effect of the antigen on the cells (**Supplementary Figure 6**). Concentrations of cytokines: IL-2, TNF-α, IFN-γ, IL-23, IL-10, IL-4, IL-12p70, IL-17A in collected cell culture supernatants were measured with MILLIPLEX^®^ Human High Sensitivity T Cell Magnetic Bead Panel (Merck KGaA, Darmstadt, Germany) on Luminex MAGPIX system according to manufacturer’s protocol. Data were analyzed using 5PL curve fit in Belysa 1.2.1 software (Merck KGaA, Darmstadt, Germany). The data on cytokine production were complemented with cytokine gene expression analysis. Briefly, RNA was isolated using a Total RNA Mini Plus kit (AA Biotechnology, Gdynia, Poland). The quality and integrity of RNA were always assessed based on non-denaturating RNA gel electrophoresis (**Supplementary Figure 7**). RNA quantity and purity were assessed using spectrophotometer BioDrop Duo (BioDrop, Harvard Bioscience, Massachusetts, USA). First strand cDNA synthesis was performed using TranScriba Kit (A&A Biotechnology, Gdynia, Poland) and reverse primers at 20 pmol concentration according to manufacturer protocol. PCR was performed using High Sensitive SYBR green mix (A&A Biotechnology, Gdynia, Poland). Gene expression was calculated using the comparative delta delta Ct method, by utilizing two housekeeping genes. The primer used and GeneBank accession number are listed in **Supplementary Table 1** (Genomed, Warsaw, Poland). For each primer pair, a curve was performed, and the effectiveness and goodness of fit R^2^ were checked. Mean Ct values for each donor, stimuli, and gene are presented in **Supplementary Table 2**. The qPCR was conducted on a Rotor-Gene Q instrument with Rotor-Gene Q series software, version 2.3.1.49 (Qiagen, Hilden, Germany). The threshold was imported from a previously prepared curve of known efficiency utilizing a single standard as the calibrator. For calculating delta delta Ct, the same donor for each gene was used as the calibrator.

#### 2.7.3 Flow cytometry analysis of cell surface markers

To assess the expression of selected surface markers on DCs the stimulated and unstimulated cells were harvested from the plates with cold PBS/2mM EDTA, washed with FC buffer (PBS/2% HI FBS, sodium azide 0.09%), and stained for 40 min. at +4 °C in dark with specific pre-titrated mouse anti-human antibodies (ELABscience, USA): anti-CD40 Elab Fluor Violet 450 (clone G28.5), anti-CD80 APC (clone 2D10), anti-CD83 PE (clone HB15e) and anti-CD86 FITC (clone BU63) or appropriate isotype control IgG1 Elab Fluor Violet 450 (clone MOPC-21), IgG1 APC (clone MOPC-21), IgG1 PE (clone MOPC-21), IgG1 FITC (clone MOPC-21). After staining cells were washed 3 times with cold FC buffer and resuspended in 0.5 ml for data acquisition. BD anti-mouse compensation beads were used according to manufacturer protocol. FC measurements were done in the Flow cytometry lab of the Faculty of Biology and Environmental Protection, University of Lodz. Data acquisition was performed on FACSymphony A1 (BD Biosciences, San Jose, CA, USA) flow cytometer, which is calibrated daily, with 405 nm laser and 450/50 bandpass filter for Elab Fluor Violet 450, 488 nm laser and 530/30 bandpass filter for FITC, 561 nm laser and 585/15 bandpass filter for PE and 637 nm laser and 670/30 bandpass filter for APC. A total of 10,000 singlet events were collected. Dublets were excluded based on FSC-A/FSC-H and SSC-A/SSC-H gates. Gating was based on CD40/CD80, CD83, CD86 cross gates. Analysis was performed using FlowJo 10.9.0 software (BD Biosciences, San Jose, CA, USA).

#### 2.7.4 Co-culture of DCs and autologous CD4^+^ T lymphocytes

For purpose of co-culture experiment immature DCs were seeded and stimulated on a 48-well plate at 1×10^5^ per well density and stimulated in the same way as the experiment described above (DC stimulation). The CD4^+^ T lymphocytes stored in liquid nitrogen were thawed and co-cultured for 48 and 96 h with the autologous DCs at a cell ratio 1:10 (1×10^5^ to 1x10^6^). A 1:1 medium volume ratio of lymphocytes to dendritic cells was used, resulting in a twofold dilution of the test protein at this stage of experiment. Cell culture supernatants were evaluated for the presence of the same cytokines as for DC stimulation Multiplex Assay. To ensure the reliability of the results, a second set of cells was cultured without lymphocytes for 48 and 96 hours, respectively. This was done to confirm which cells were responsible for producing which cytokines. The data on cytokine production were also complemented with cytokine gene expression analysis as described above.

All results from the cytokine multiplex are presented in the **Supplementary Material**, **Supplementary Figure 3**.

In the cell co-culture experiment, an additional control was used in which CD4^+^ lymphocytes were cultured alone in the presence of either the rROP8 antigen at the same concentrations as in the actual experiment. The cytokine levels in the cell culture medium were determined using the Multiplex assay, as in the actual experiment.

**Figure 2:**
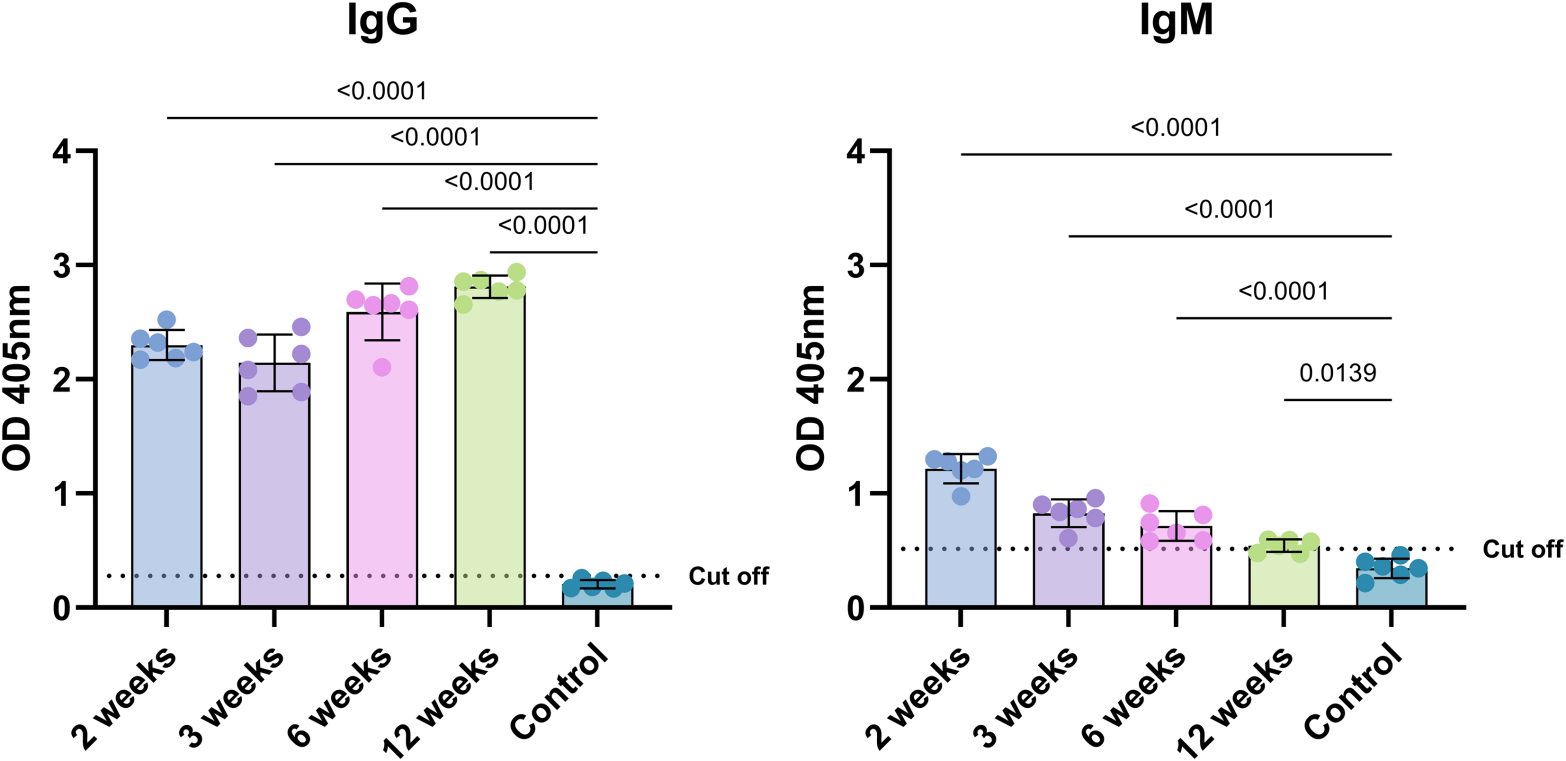
*T. gondii* DX infected mice serum IgG and IgM class antibodies reactivity with rROP8 antigen. Values presented as mean and standard deviation (n = 6 mice). Analysis was performed using ordinary one-way ANOVA followed by Dunnett`s multiple comparisons test to compare each group with the control. The dotted line represents the calculated cut-off value (control mean + 2*SD).

### 2.8 ROP8 as a thiazolidin-4-one derivatives therapeutic target

#### 2.8.1 Chemistry and compound preparation

All thiazolidin-4-one derivatives namely (4-oxothiazolidin-5-yl)acetic acid and (4-oxothiazolidin-5-ylidene)acetic acid derivatives were synthesized by our research group previously. The methods of synthesis, physicochemical characteristics details of compounds **82–88** and **91-95**, and concentration of those compounds required for 50% inhibition of *T. gondii* proliferation *in vitro* (IC_50_) were presented in our previously published paper (22) All compounds were dissolved in dimethyl sulfoxide (Merck KGaA, Darmstadt, Germany) to 0.5 mM. All compounds were freshly prepared before the experiment and sterile filtered.

#### 2.8.2 Drug affinity responsive target stability assay

Drug affinity responsive target stability (DARTS) is a robust method for the detection of novel small molecule protein targets. This assay is based on the stabilization of the target protein that occurs upon small molecule binding by detecting the binding-induced increase in resistance to proteolysis and was performed as previously described with modification (23). The rROP8 protein was diluted in TNC buffer to 2.5 μg/μL. TNC buffer (1 mL, 10×) 500 μL 1 M Tris-HCl pH 8.0, 100 μL 5 M NaCl, 100 μL 1 M CaCl_2_, and 300 μL ultrapure water. Then, to 20 μL of rROP8, 0.2 μL of 0.5 mM compounds 82–88 and 91-95, was added. As a control, 0.2 μL of DMSO (vehicle control) was used. Samples containing the protein and compound were incubated for 1 h at room temperature with shaking. Subsequently, pronase (10 mg/mL, Roche) dilution 1:400 was prepared in TNC buffer. After incubation with (4-oxothiazolidin-5-yl)acetic acid or (4-oxothiazolidin-5-ylidene)acetic acid derivatives or DMSO, 2 μL of pronase solution was added. For the nondigested sample, 2 μL of 1×TNC buffer instead of protease was used. Samples were incubated at room temperature with the pronase for 15 min. The digestion reaction was stopped by adding 2 μL of 20×EDTA-free protease inhibitors and incubating on ice for 10 min. Then, SDS–PAGE electrophoresis was performed, gels were stained with Imperial™ Protein Stain (Thermo Fisher Scientific, Waltham, MA, USA) and the intensity of the bands was quantified using ImageJ software (National Institutes of Health, Bethesda, MD, USA).

#### 2.8.3 Influence of compounds during *Toxoplasma* growth

Hs27 cells were cultured on Lab-Tek 4-well Chamber Slides (Nunc) (5×10^4^ cells/500 µl per well). After 48 h, the medium was removed and then 5×10^5^/500µL/well tachyzoites of the RH-GFP strain were added to the cell monolayers, in parasite culture medium for 1 h. Then, the cells were washed to remove extracellular parasites, and the cells were treated with the compounds **82–88** and **91–95** at IC_50_ concentration for 24 h. As the controls, Hs27 cells were infected, but not treated. After subsequent 24h of incubation, the slides were washed with sterile PBS, fixed with formaldehyde solution (Merck KGaA, Darmstadt, Germany), 3.7% in PBS pH 7.4 for 15 min, and stained with DAPI for 2 minutes (1mg/ml, ThermoFisher Scientific, Waltham, MA) and Texas Red-X Phalloidin for 40min (1.65mM; Thermo Fisher Scientific, Waltham, MA, USA). Cells were visualized using a confocal scanning laser microscope, Leica TCS SP8, 63x/1.40 oil, equipped with Leica LAS AF Lite Software for image processing. Three independent experiments on triplicate chamber slides using the same conditions were performed.

### 2.9 Statistical analysis

Graphs and all statistical analyses were performed using GraphPad Prism 10.2.2 for Windows (Dotmatics, GraphPad Software, California, USA). All data are presented as mean and standard deviation (SD). The Shapiro-Wilk and D’Agostino-Pearson tests were used to assess the Gaussian distribution of the data, residuals or differences, along with analysis of the Q-Q plots. The Brown-Forsythe test was used to test the equality of the group variances in ANOVA model. In order to compare three or more groups, an ANOVA analysis was conducted, and, alternatively, a Kruskal-Wallis test was employed for data that did not meet the assumptions of normality and heteroscedasticity. Receiver operating characteristics (ROC) analysis was performed to obtain the area under the curve (AUC), ROC-cut off, the sensitivity, and the specificity of the test. In order to determine the correlation between the ELISA values for each human serum sample obtained using TLA and rROP8, a Pearson correlation analysis was conducted. A two-sample t-test was employed to compare control and rROP8 values in cell stimulation experiments. A F-test was used to test equality of variances. Data obtained on human donor dendritic cells cultures were analyzed using a ratio paired t-test. A two-way repeated measures (RM) ANOVA was employed to compare co-cultures at 48 and 96 hours, with the assumption of sphericity. Similarly, RM ANOVA and RM two-way ANOVA were employed for the examination of ratios of control/rROP8 values. Relative band intensity was analyzed using ordinary one-way ANOVA. Correlation between relative band intensity and IC50 values was analyzed using rho spearman analysis. The *post hoc* test used for each data analysis is provided below the figures. In all analytical procedures, the alpha value was set at 0.05.

## 3 Results

### 3.1 Diagnostic utility of recombinant rROP8 antigen

Since there is a need to constantly improve available diagnostic methods of toxoplasmosis, continuous research on the use of recombinant parasite proteins is being conducted. Thus, as a part of a thorough characterization of ROP8 the protein was also evaluated for its potential diagnostic utility. To assess the usefulness of the rROP8 protein as a diagnostic tool for *T. gondii* infection, a series of experiments were performed. The first was to assess the ability of the protein to detect specific IgG and IgM class antibodies in the sera of *T. gondii* experimentally infected mice, using the ELISA test (**Figure 2**). The rROP8 protein allowed the detection of IgG class antibodies in the animals at any time post-infection, even after only 2 weeks and in each case, the test mean result was significantly higher than the mean of the control group of uninfected animals. This protein also allowed the detection of IgM class antibodies in every individual in the groups 2, 3 and 6 weeks after infection, with values above the cut-off line, whereas in the 12-week group this was only possible in 3 out of 6 individuals, nevertheless the mean values of each group were still significantly higher than those of the control group.

**Figure 3:**
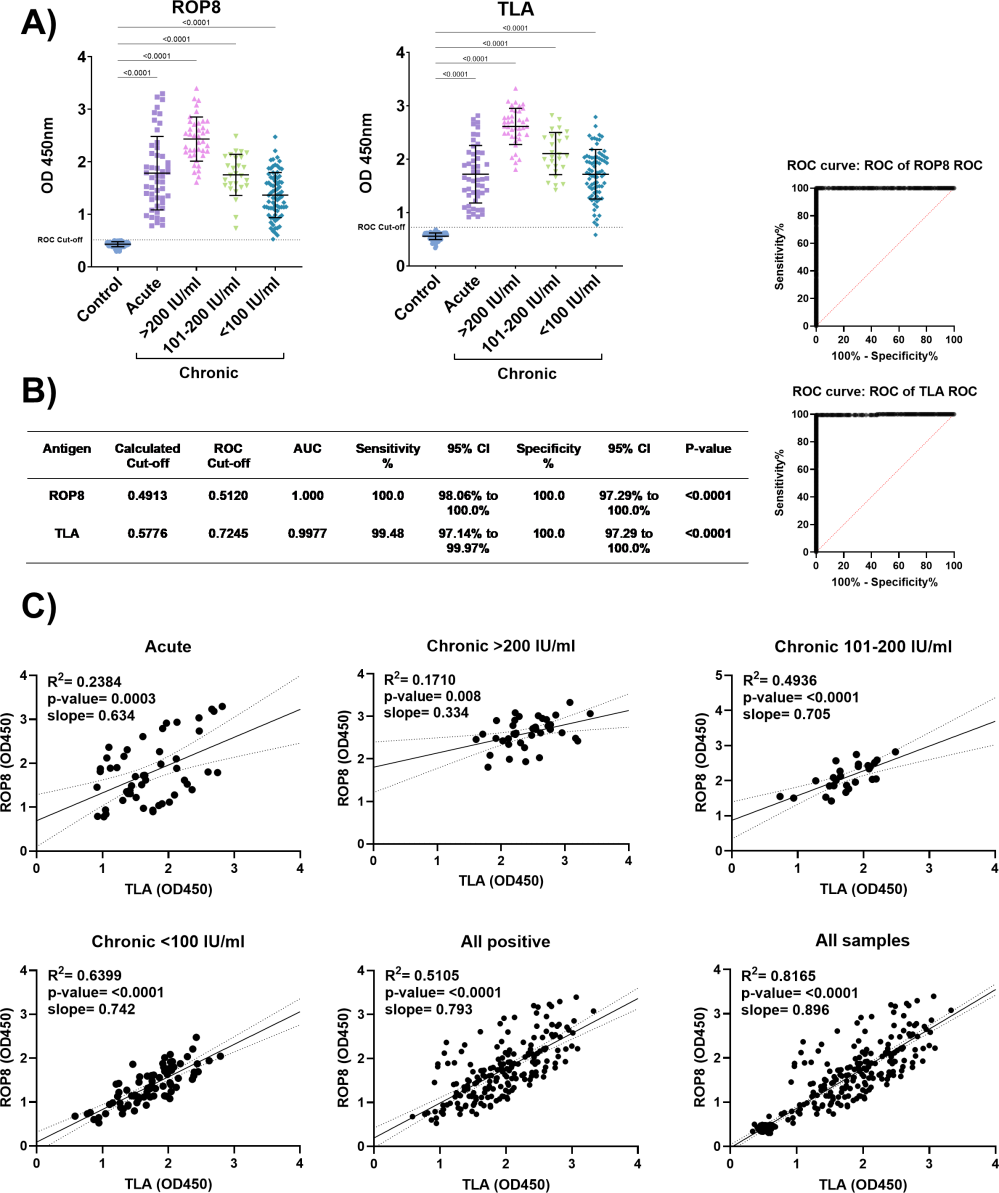
Serodiagnostic utility of rROP8 antigen. **(A)** Human sera IgG class antibody reactivity with rROP8 antigen. Serum samples were divided into groups acute (n = 50), chronic (> 200 IU/ml n = 40, 101–200 IU/ml n = 27, < 100 IU/ml n = 77) or control uninfected (n = 138). Analysis was performed using the Kruskal-Wallis test followed by Dunn`s multiple comparisons test to compare each group with the control. The adjusted p-value is shown above brackets. **(B)** Results of ROC analysis. **(C)** Scatter plot of TLA and rROP8 results for each individual, with linear regression fit in with 95% CI. Analysis performed using Pearson r-test.

The antigen has also been used to detect IgG class serum antibodies in sera of humans infected with *T. gondii* at different stages of the disease, which was confirmed using commercially available tests. To compare the actual usefulness of the rROP8 protein, the same serum samples were tested with TLA, a lysate of tachyzoites, a preparation used in current diagnostic tests (**Figure 2**).

The rROP8 protein correctly classified all the infected patients as positive using the ROC analysis cut-off of 0.512, with an estimated test sensitivity of 100% (98.06% - 100% CI 95%) and specificity of 100% (97.29% - 100% CI 95%) (**Figures 3A, B**). A mixture of native water-soluble antigens, TLA correctly detected almost all samples with a sensitivity of 99.48% (97.14% - 99.97% CI 95%) and a specificity of 100% (97.29% - 100% CI 95%).

Raw test results for a given patient obtained using rROP8 protein and TLA correlate significantly in a linear manner, with a slope ranging from 0.334 to 0.896 (**Figure 3C**). The best fit is observed in the case of people with chronic toxoplasmosis, at the initial stage with an antibody titer of <100 IU/ml, while the worst fit is observed in the case of samples with a titer of >200 IU/ml, here we can also see a decrease in the slope of the line by more than half compared to others.

In summary, rROP8 has been demonstrated to exhibit a high capacity to bind IgG antibodies that occur naturally during *T. gondii* infection in both humans and experimentally infected animals. For both types of serum, the reactivity of IgG antibodies to the rROP8 antigen is comparable to that obtained for the native TLA antigen. Conversely, rROP8 exhibited inferior IgM antibody recognition properties in comparison to TLA. Further studies are required to validate rROP8 as an antigen for potential diagnostic use.

### 3.2 Monocyte and macrophage activation assays

**Figure 4:**
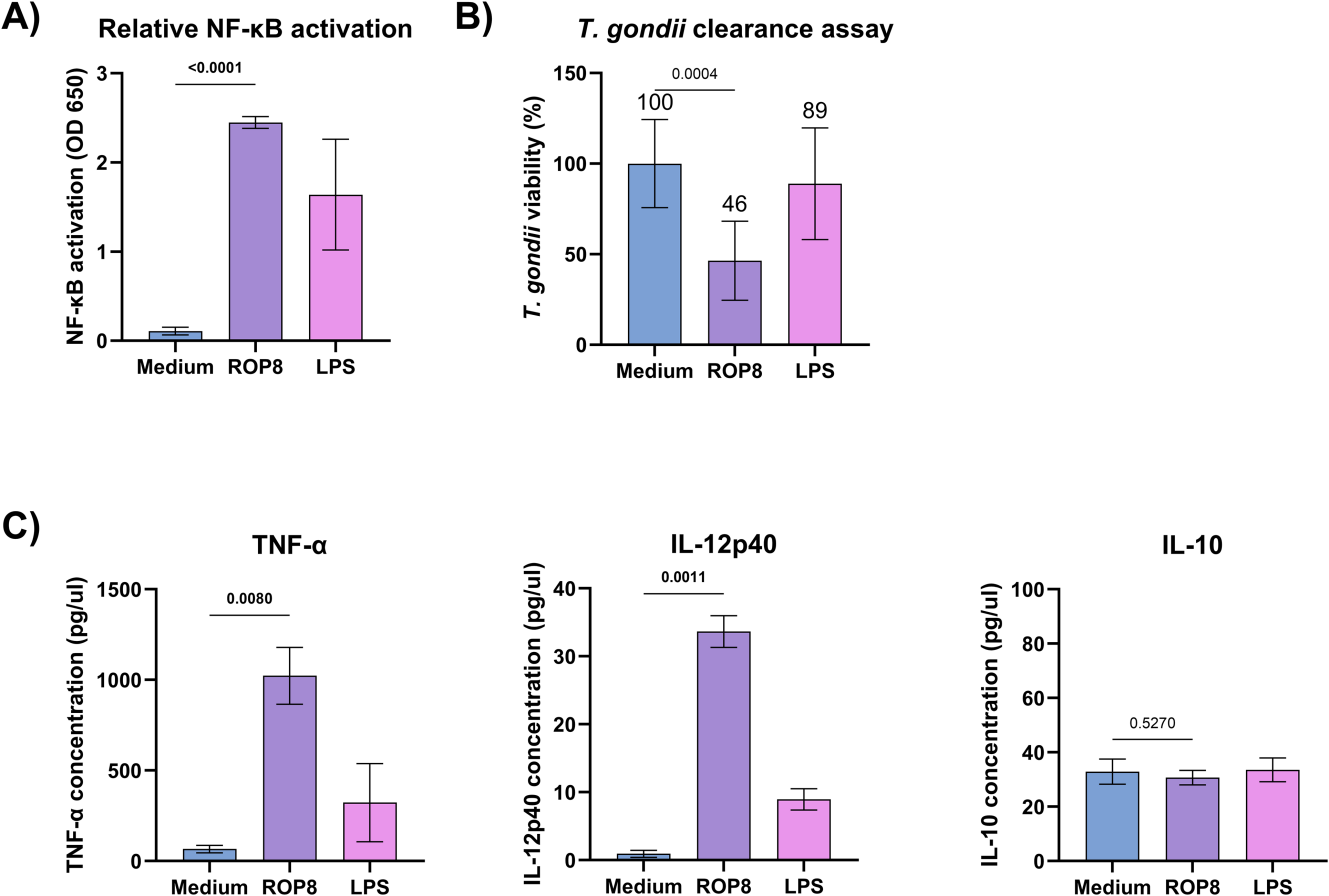
*In vitro* stimulation of human monocytes and mouse macrophages **(A)** Relative activation of the NF-κB pathway (OD 650 nm) after stimulation of THP1-Blue monocytes with rROP8, LPS (positive control) or unstimulated cells (medium). Data are presented as mean and SD (n = 6 independent experiments). **(B)***T. gondii* clearance assay shows *T. gondii* RH-GFP viability in rROP8 antigen, LPS (control) stimulated ANA1 macrophages or unstimulated cells (medium). Data are presented as mean and SD (n = 8 independent experiments). **C)** Selected cytokines production levels by ANA1 macrophages after stimulation with rROP8 antigen, LPS (control), or unstimulated cells (medium). Data are presented as mean and SD (n = 3 independent experiments). Data presented in graphs **A–C)** were analyzed using the unpaired t-test with Welch`s correction to compare the medium with rROP8. The two-tailed p-value is shown above brackets.

Due to the need to improve available anti-*T. gondii* immunoproxilaxis for animals and develop one dedicated to humans, the next step was characterization of immune potential of the ROP8 recombinant protein as an indication of its possible future incorporation into experimental vaccine design based on selected highly immunogenic parasite proteins. The *in vitro* screening of antigens is indispensable for immunoprophylaxis studies to narrow down an almost infinite number of parasite antigens combinations for *in vivo* evaluations. In order to evaluate the immunogenic potential of the rROP8 protein, a series of experiments were conducted to stimulate the innate immune cells responsible for antigen recognition and signal transduction, with the aim of facilitating the development of adaptive immune mechanisms. To achieve this objective, human THP1-Blue line cells with the NF-κB inflammatory pathway activation reporter gene were employed (**Figure 4A**). Furthermore, mouse macrophages of the ANA1 line were utilized to evaluate the profile of cytokines secreted following stimulation (**Figure 4C**), as well as the level of phagocytic activity of the cells after stimulation (**Figure 4B**).

Following each experiment, a cytotoxicity test based on resazurin reduction was conducted to exclude any toxic effects of the protein on the cells, which could have interfered with the results (**Supplementary Figure 1A**). Additionally, human monocyte stimulation tests were performed using uninduced *E. coli* (Rosetta(DE3)pLysS) lysate, which was produced and purified in conditions similar to that of the actual protein, without the endotoxin removal step (**Supplementary Figure 1B**). This approach enabled the validation of potential false positives.

The rROP8 protein exhibited a pronounced capacity to activate the NF-κB pathway in human monocytes, exhibiting a markedly elevated level of stimulation (p < 0.0001) in comparison to the unstimulated baseline. Additionally, the protein stimulated the production of cytokines by mouse macrophages, such as TNF-α and IL-12p40. These stimulated cells exhibited a significantly higher production of these cytokines in comparison to the unstimulated cells, with p-values of 0.008 and 0.001, respectively. Following stimulation, mouse macrophages of the ANA1 line exhibited markedly enhanced phagocytic activity, enabling the clearance of the parasite from the culture and, consequently, a significant (p=0.004) reduction in its numbers by up to 46% in comparison to non-stimulated cells.

### 3.3 DCs activation and co-culture with T-helper lymphocytes

It is challenging to ascertain the extent to which APC cell activation will result in an efficacious lymphocyte response. In light of these considerations, a series of experiments have been proposed that combine the stimulation of APC cells with the subsequent signal transduction to helper lymphocytes, to assess their activation and, consequently, their polarization of response. This approach offers a comprehensive and detailed insight into the immunogenicity of a given antigen, providing plenty of information that can be utilized to enhance our understanding of the underlying mechanisms.

For this purpose, human monocytes were obtained from healthy blood donors who were negative for *T. gondii* infection. Thereafter, dendritic cells were derived from the monocytes and stimulated with the rROP8 protein.

**Figure 5:**
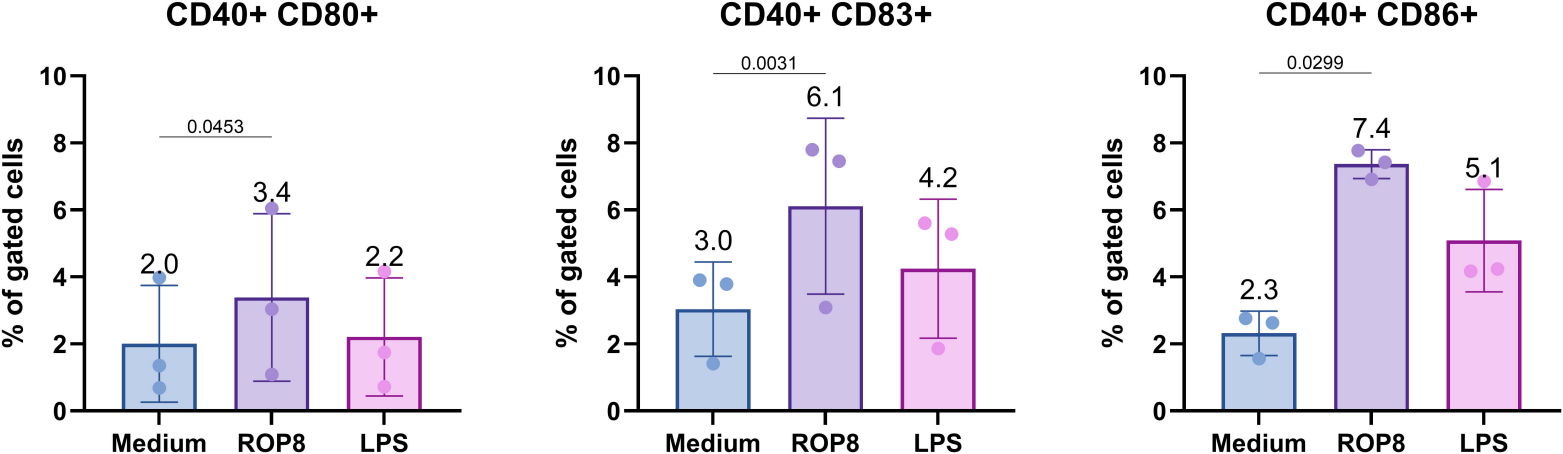
Flow cytometry analysis of stimulated DC cells. Percentage of positive gated cells with the CD40, CD80, CD83 and CD86 markers expression on DC cells surface, after 24h stimulation with rROP8, LPS (positive control) and unstimulated (medium). Data presented as mean (value above bar) and standard deviation (n = 3 donors) were analyzed using the ratio paired t-test to compare medium with rROP8. The two-tailed p-value is shown above brackets.

Twenty-four hours after the stimulation of cells with rROP8 antigen, the deposition of DC cell activation and maturation markers, including CD80, CD83 and CD86, was estimated by flow cytometry (**Figure 5**). The data demonstrate a notable elevation in the proportion of positive CD40^+^ CD83^+^ (p = 0.0031) and CD40^+^ CD86^+^ (p = 0.0299) cells, exhibiting a twofold and nearly threefold increase, respectively, in comparison to non-stimulated cells. The smallest increase is observed for CD40^+^ CD80^+^ cells, with a 1.7-fold increase, which nevertheless remains significant (p = 0.0453). It is also worth noting that the difference between stimulated and non-stimulated cells is homogeneous between donors.

**Figure 6:**
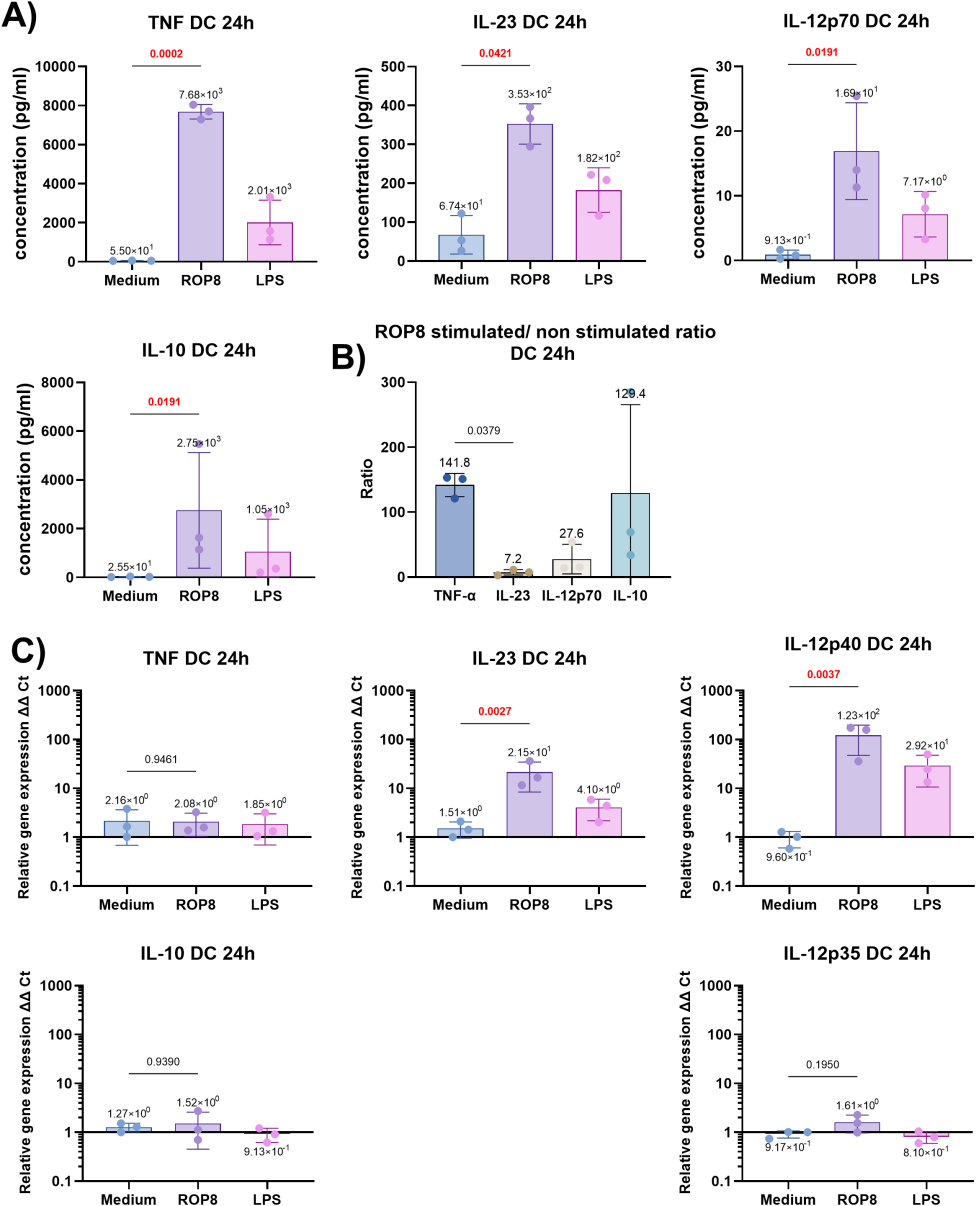
Dendritic cells cytokine response. **(A)** Selected cytokine production levels of DC cells stimulated for 24 h with rROP8 antigen, LPS (positive control), or unstimulated (medium). Data presented as mean (value above bar) and standard deviation (n = 3 donors) were analyzed using the ratio paired t-test to compare medium with rROP8. The two-tailed p-value is shown above brackets. P-values < 0.05 are highlighted in red. **(B)** rROP8 stimulated/non-stimulated cells cytokine value ratio. Data presented as mean (value above bar) and standard deviation (n = 3 donors). Analysis was performed using RM one-way ANOVA followed by Sidak multiple comparisons test, to calculate every possible comparison. The adjusted p-value is shown above brackets. Only comparisons with p-value < 0.05 are shown. **(C)** Relative expression of selected cytokine genes in DC cells stimulated for 24h with rROP8 antigen, LPS (positive control), or unstimulated (medium). Data presented as mean (value above bar) and standard deviation (n = 3 donors) were analyzed using the ratio paired t-test to compare medium with rROP8. The two-tailed p-value is shown above brackets. P-values < 0.05 are highlighted in red.

To gain further insight into the response, the concentrations of cytokines, which play a pivotal role in the context of the APC cell response, and the expression of related genes were determined (**Figure 6**). The production of pro-inflammatory cytokines, including TNF-α (p = 0.0002), IL-23 (p = 0.0421) and IL-12p70 (p = 0.0191), was significantly elevated in stimulated cells (**Figure 6A**). The excessive secretion of inflammatory cytokines probably contributed indirectly to anti-inflammatory response by the production of significant level of IL-10 (p = 0.0191), however with a notable discrepancy observed between donors. The rROP8 ratio of stimulated/unstimulated cells indicates that the most notable mean change is observed for TNF-α, followed by IL-10, IL-12p70 and finally IL-23, which exhibited a significantly lower ratio than TNF-α (p = 0.0379) (**Figure 6B**). Gene expression of these cytokines, 24 hours post stimulation, is noticeable for IL-23 and IL-12p40, while not for IL-12p35, TNF-α or IL-10 (**Figure 6C**).

**Figure 7:**
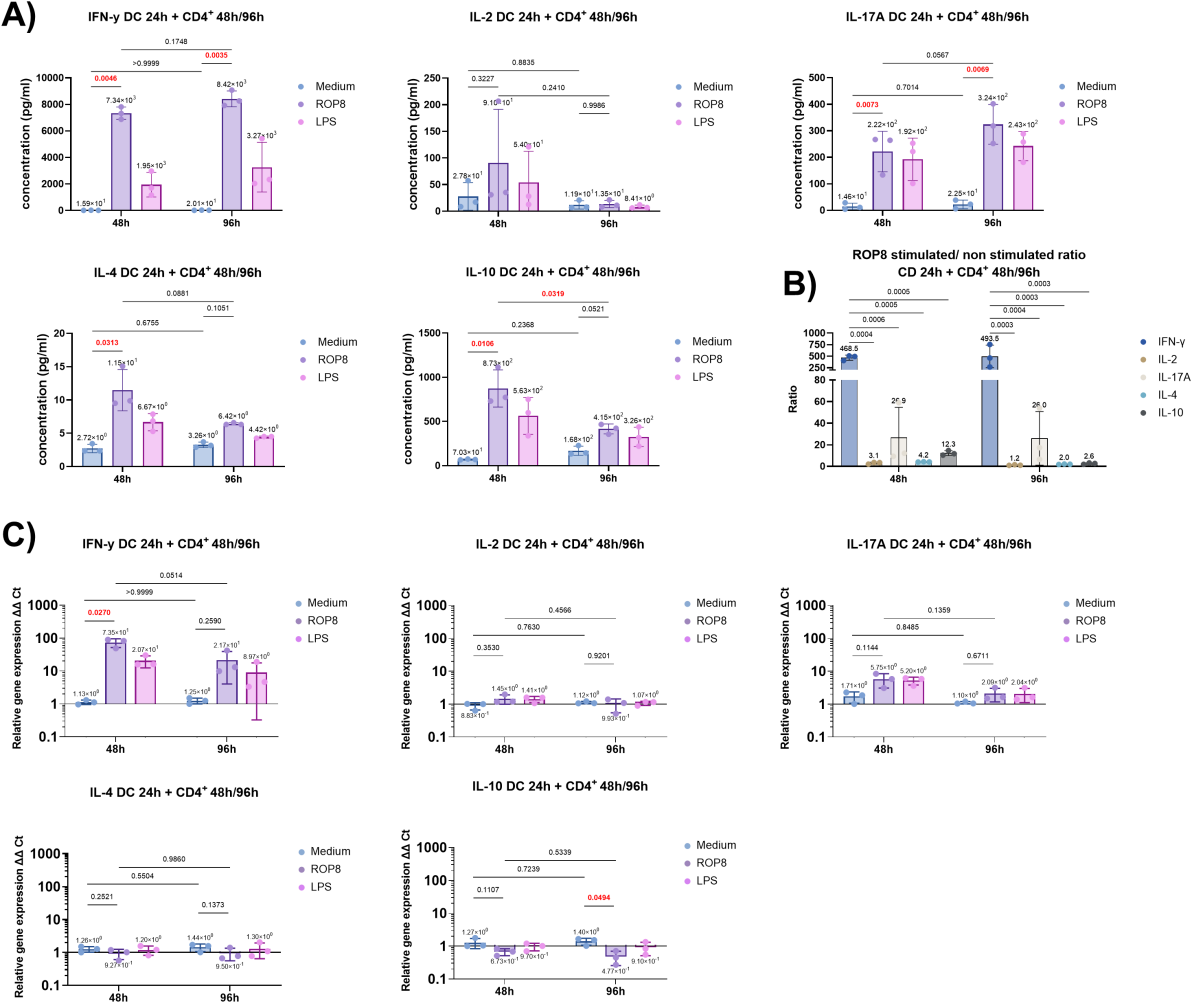
Cytokine response in cultures of dendritic cells with CD4+ helper lymphocytes. **(A)** Selected cytokines production levels by CD4^+^ lymphocytes after 48 and 96 hours co-culture with DC cells, previously stimulated with rROP8 antigen, LPS (positive control), or unstimulated (medium) for 24 h. Data presented as mean (value above bar) and standard deviation (n = 3 donors). Data were analyzed using the RM two-way ANOVA followed by Holm-Sidak multiple comparisons test, to compare unstimulated (medium) cells with rROP8 stimulated values in and between time frames. The adjusted p-value is shown above brackets. P-values < 0.05 are highlighted in red. **(B)** rROP8 stimulated/non-stimulated cells cytokine value ratio. Data presented as mean (value above bar) and standard deviation (n = 3 donors). Analysis was performed using RM two-way ANOVA followed by the Holm-Sidak multiple comparisons test, to calculate every possible comparisons. The adjusted p-value is shown above brackets. Only comparisons with p-value < 0.05 are shown. **(C)** Relative expression of selected cytokine genes in DC cells stimulated for 24 h with rROP8 antigen, LPS (positive control), or unstimulated (medium) and then cultured with CD4^+^ lymphocytes for 48 and 96 hours. Data presented as mean (value above bar) and standard deviation (n = 3 donors).

Following a 24-hour period of stimulation of DC cells with rROP8 protein, previously isolated CD4^+^ lymphocytes from each donor were introduced to the culture and cells were co-cultured for 48 and 96 hours. Subsequently, the concentrations of cytokines, that are crucial for the response of helper lymphocytes of the Th1, Th2 and Th17 phenotype and the expression of their genes were examined (**Figure 7**). The lymphocyte response to rROP8 DC stimulated cells was observed to be significant, with the production of high levels of IFN-γ occurring both after 48 (p = 0.0035) and 96 (p = 0.0035) hours (**Figure 7A**). However, while there was a discernible tendency for this response to increase over time, it did not reach statistical significance (p = 0.1748). A comparable outcome was noted for IL-17A production following a 48-hour (p = 0.073) and 96-hour (p = 0.0069) period. IL-2 production exhibited a threefold increase after 48 hours; however, statistical significance was not attained due to the considerable discrepancy between donors. Furthermore, after 96 hours, no visible differences were observed between stimulated and unstimulated cells.

Additionally, anti-inflammatory cytokines concentrations, including IL-4 and IL-10, were markedly produced following rROP8 stimulation, with p-values of 0.00313 and 0.0106, respectively. However, the distinction between rROP8-stimulated and unstimulated cells diminished after 96 hours, with a particularly significant decline observed in IL-10 production (p = 0.0319).

The ratio of rROP8-stimulated cells to unstimulated cells demonstrates that the IFN-γ response was dominant and statistically significant over the other cytokines, with a ratio of approximately 480-fold mean concentration change (**Figure 7B**). This is followed by IL-17A, with a ratio of approximately 26.5.

The gene expression of the studied cytokines was found to be consistent with the observed protein production (**Figure 7C**). A notable increase in the relative gene expression level of INF-γ was observed following 48 hours of co-culture (p = 0.0270, approximately 70-fold), which was not statistically significant after 96 hours (approximately 20-fold). The decrease between 48 hours and 96 hours was approaching statistical significance (p = 0.0514). The effect observed in the case of IL-17A was relatively minor, with an approximate 4-fold change in gene expression after 48 hours, which was not statistically significant (p = 0.1144), and a 2-fold change after 96 hours. The interleukin 2 gene was also weakly expressed after 48 hours of co-culture, with a non-significant 1.5-fold increase after 48 hours and no change after 96 hours. No discernible elevation in IL-4 and IL-10 gene expression was observed following 48 and 96 hours of co-culture. However, it was noted that IL-10 expression in rROP8 stimulated cells was comparatively diminished relative to unstimulated cells after 48 hours, with a particularly pronounced decline evident after 96 hours (p = 0.0494).

In summary, the recombinant ROP8 protein has been demonstrated to possess the capacity to activate human THP-1 monocytes by means of NF-κB pathway activation. The study also revealed that the antigen leads to the production of inflammatory cytokines such as TNF-alpha and IL-12 in ANA-1 mouse macrophages. Furthermore, it was observed that the antigen enhanced the ability of these cells to combat *T. gondii* following prior stimulation. Moreover, it has been demonstrated that following the stimulation of human dendritic cells, a robust pro-inflammatory response is elicited, resulting in the polarization of T helper lymphocytes towards the Th1/Th17 phenotype.

### 3.4 ROP8 as a potential drug target

**Figure 8:**
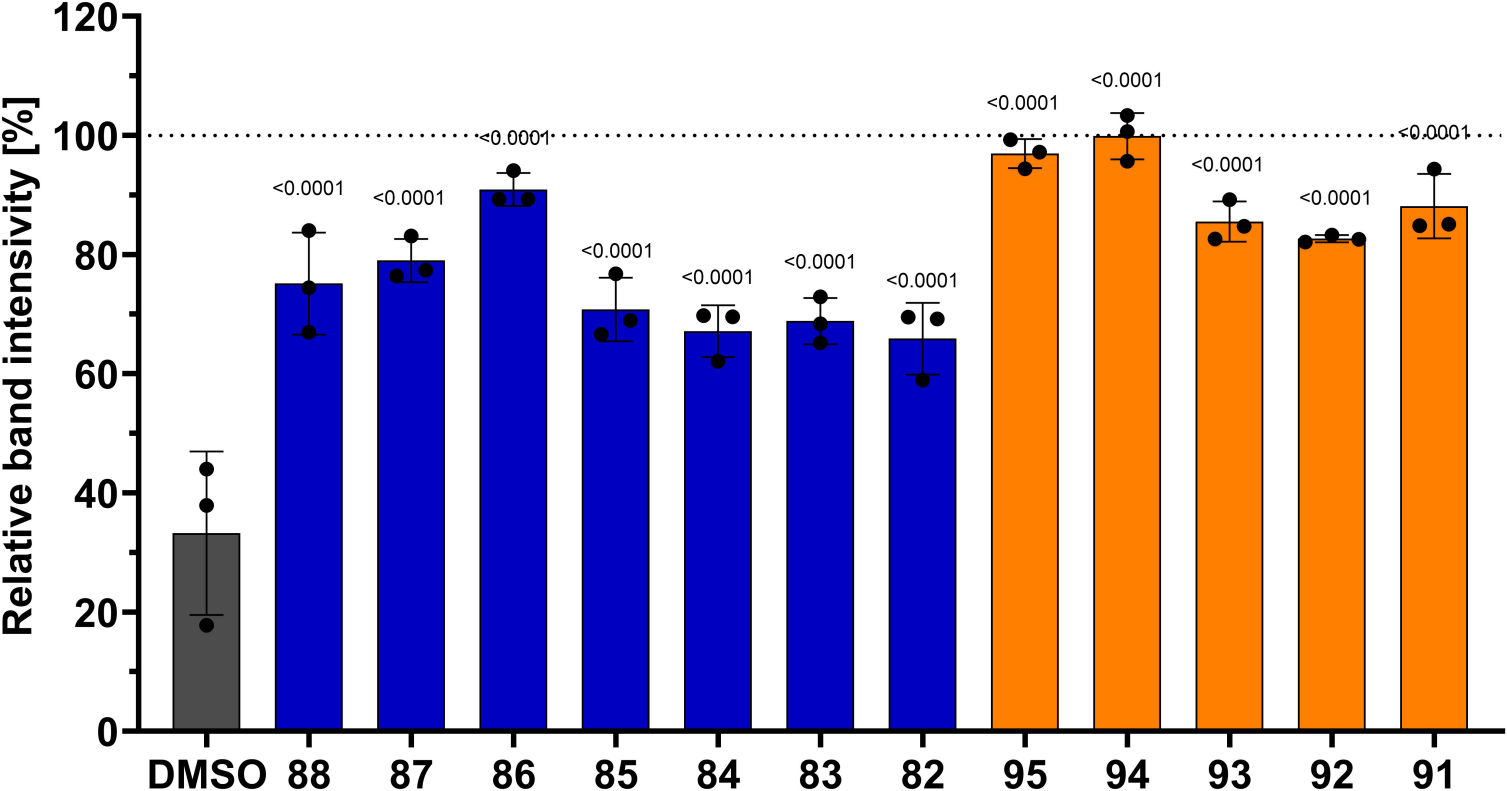
Protection of rROP8 from proteolysis by: (4-oxothiazolidin-5-ylo)acetic acid derivatives (blue) (82: R = 4-Cl, R_1_ = 3-OH; 83: R = 4-Cl, R_1_ = 4-OH; 84: R = 4-Cl, R_1_ = 3-OCH_3_, 4-OH; 85: R = 4-Cl, R_1_ = 3-OC_2_H_5_, 4-OH; 86: R = 4-Cl, R_1_ = 3-Cl, 4-OH; 87: R = 4-Cl, R_1_ = 3-Br, 4-OH; 88: R = 2,4-diCl, R_1_ = 3-OCH_3_, 4-OH) and (4-oxothiazolidin-5-ylidene)acetic acid derivatives (orange) (91: R=H, R_1_ = 4-OH; 92: R=H, R_1_ = 3-OCH_3_, 4-OH; 93: R=H, R_1_ = 3-OC_2_H_5_, 4-OH; 94: R=H, R_1_ = 3-Cl, 4-OH; 95: R=H, R_1_ = 3-Br, 4-OH) in the presence of 5 µM compound and with the 1:400 pronase:protein ratio. Analysis was performed using one-way ANOVA with Holms-Sidak multiple comparisons test, to compare each compound with DMSO value. The adjusted p-value is shown above bars. Values are presented as mean and standard deviation (n = 3 independent experiments).

The last part of the study focused on ROP8 in terms of *T. gondii* treatment that also requires improvements and new compounds with potential antiparasitic activity are being tested in many laboratories for possible application. Trotsko et al. previously designed and synthesized a series of thiazolidin-4-one derivatives, which we subsequently assessed for their ability to inhibit the *in vitro* proliferation of the *T. gondii* parasite. Our findings indicate that all previously designed active compounds in the series demonstrate superior inhibition of *T. gondii* tachyzoite proliferation compared to reference drugs (22). Literature data indicate that derivatives belonging to that group of compounds can exhibit an inhibitory effect on the *T. gondii* ROP8 protein. Thus, we selected the most potent anti-*T. gondii* compounds from the previous study to further investigate the mechanism underlying their potency and we conducted a drug affinity responsive target stability (DARTS) assay to assess the interaction of these compounds with the rROP8 protein. Incubation with selected thiazolidin-4-one derivatives protected the rROP8 protein in comparison to the vehicle control (DMSO). The SDS-PAGE results demonstrated the presence of both non-digested rROP8 protein and a degraded form at ratios of 1:400 pronase to protein. As shown in **Figure 8**, the proteolytic degradation of rROP8 was significantly (p<0.0001) inhibited by the presence of thiazolidin-4-one derivatives, especially (4-oxothiazolidin-5-yl)acetic acid compound (86) and (4-oxothiazolidin-5-ylidene)acetic acid derivatives (91–95), resulted in a notable reduction in proteolytic activity. These results underscore the ability of these derivatives to protect rROP8 from proteolytic cleavage, particularly at the concentration of 5 µM. The SDS–PAGE results for rROP8 from the drug affinity responsive target stability (DARTS) assay are presented in the **Supplementary Materials** (**Supplementary Figure 2**).

**Figure 9:**
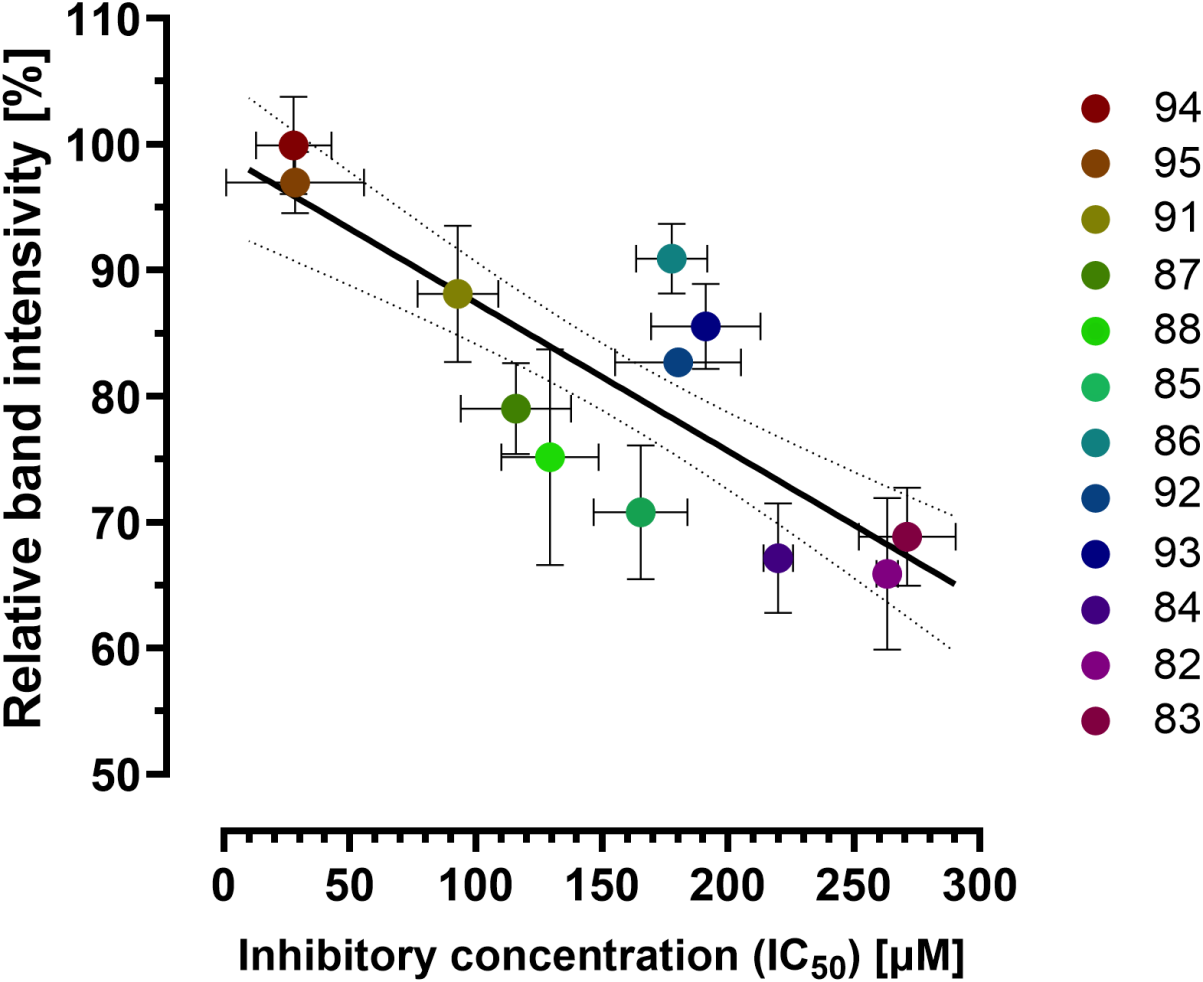
The correlation between the percentage of relative band intensity from DARTS assay and the concentration of the compound required for 50% inhibition of *T. gondii* proliferation *in vitro* (IC_50_) for compounds 82-86 (4-oxothiazolidin-5-yl)acetic acid compound and 91–95 (4-oxothiazolidin-5-ylidene)acetic acid derivatives (r=-0.76; p=0.0031). Data presented as mean and standard deviation The statistical relationships were analyzed using Spearman correlation.

Building on the obtained results, further analysis was conducted to investigate the relationship between the inhibitory effects on the rROP8 protein and the compounds’ ability to inhibit *T. gondii* proliferation *in vitro*. The results of the drug affinity responsive target stability (DARTS) assay show a strong negative correlation with the concentration of the compound required for 50% inhibition of *T. gondii* proliferation *in vitro* (IC_50_, data from (22)). This correlation is statistically significant, with a Spearman correlation coefficient of r=−0.76 and p=0.0031 (**Figure 9**). The analysis includes both (4-oxothiazolidin-5-yl)acetic acid and (4-oxothiazolidin-5-ylidene)acetic acid derivatives. These findings suggest that the anti-*T. gondii* efficacy of thiazolidin-4-one derivatives is associated with their inhibitory effects on the rROP8 protein.

**Figure 10:**
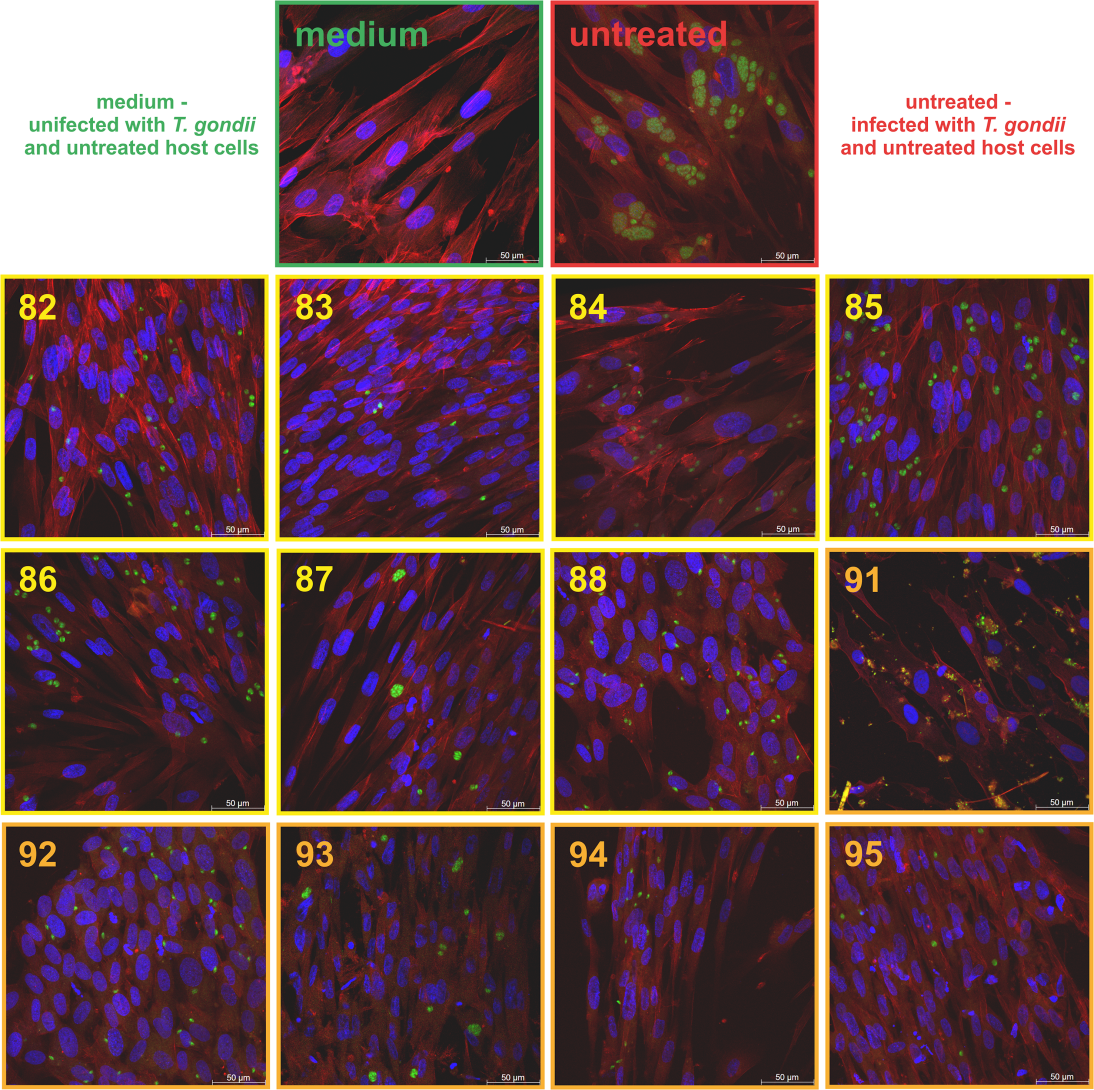
Representative images of confocal microscopy showing inhibition of parasitophorous vacuole formation and disruption of tachyzoites division in host cells (Hs27) after 24h incubation with the tested compounds at IC_50_; *T. gondii* RH-GFP (green), host cells stained with Texas Red-X Phalloidin (red), host cells nuclei stained with DAPI (blue).

The ROP8 protein is involved in the formation of parasitophorous vacuoles (9), which are critical for tachyzoite proliferation and the subsequent spread of the parasite. The objective of this test was to assess whether the compounds under investigation inhibit vacuole formation and effectively block tachyzoite division. The results of the microscopic analysis (**Figure 10**) demonstrate a reduction in parasitophorous vacuole formation compared to untreated cells, as well as a decrease in the number of tachyzoites per vacuole. These findings suggest that the compounds disrupt the ability of *T. gondii* to proliferate within host cells.

In summary, the results of the present study demonstrated that (4-oxothiazolidin-5-yl)acetic acid compounds and (4-oxothiazolidin-5-ylidene)acetic acid derivatives, which have been shown to possess high anti-*T. gondii* activity in a study previously published, also exhibited the capacity to bind the *T. gondii* ROP8 protein *in vitro* in the DARTS assay. Furthermore, the binding strength exhibited a positive correlation with the IC50 values of the anti-*T. gondii* activity. The results of the *in vitro* imaging assay suggest that the tested compounds may affect the ability of *T. gondii* to proliferate within the host cell.

## 4 Discussion

The protozoan parasite *T. gondii* remains a subject of intensive research, focusing on three key areas: diagnostics, immunoprophilaxis, and treatment. The diagnostic of toxoplasmosis is primarily based on serological methods, with commercially available tests relying on native TLA proteins. However, there are limitations to this approach, including the high cost of culturing the parasite and the impact of culture quality on the antigenic composition of the lysate, which can result in significant batch-to-batch variation. In light of these challenges, researchers globally are exploring alternatives, such as recombinant proteins, which offer a cost-effective and more defined solution, that may also help to distinguish between stages of infection (16, 24). The field of immunoprophylaxis has been the subject of extensive research, given the lack of a vaccine suitable for human use and the fact that the only commercially available Toxovax™ vaccine is based on an attenuated strain. This vaccine is used mainly to reduce the incidence of abortion in sheep, which limits financial losses (25). However, it is not suitable for human use. The parasite itself has a complex life cycle, as previously mentioned. This is one of the reasons why it is challenging to develop an effective immunoprophylaxis. However, other factors, including the parasite’s mechanisms to evade the immune response, are also a major problem. Most of the proposed new vaccine approaches are based on the use of single or multiple antigens of the parasite in the form of recombinant proteins, DNA or RNA vaccines. Wide variation of different proteins present throughout the parasite’s life cycle represents a significant challenge in the selection of vaccine candidates (26, 27). Narrowing down the number of potential vaccine candidates could prove beneficial in the research for an effective vaccine. The last-mentioned problem associated with *T. gondii* infection is its treatment, which is usually based on pyrimethamine and sulfadiazine. Available treatment options rely on drugs, which cause numerous side effects and are efficient only toward fast-dividing tachyzoite stage associated with acute infection. Thus, researchers are investigating new compounds with anti-*T. gondii* activity that will have greater efficacy and selectivity than those available. This is also a timely topic in light of reports of *T. gondii* resistance and its mechanisms to available drugs (28).

The experiments and results presented in this study were designed to assess the utility of the rROP8 protein for the diagnostics of toxoplasmosis, to determine its *in vitro* immunogenic potential, and to evaluate this antigen as a potential molecular target for new chemotherapeutics. The protein was obtained in a prokaryotic expression system, purified by metal affinity chromatography, and used for further studies. The initial study was to assess the antigenicity of the protein. Bioinformatics analyses classify the ROP8 antigen as antigenic, with multiple proposed linear and conformational epitopes for B cells described in the literature, considering indexes such as surface accessibility, flexibility, hydrophilicity, antigenicity, turns, exposed surface, polarity, antigenic propensity (11). The ability of the recombinant antigen to recognize antibodies produced against the native protein provides valuable information about its potential for use in diagnosis and furthermore, its immunoprophylactic potency, suggesting that upon immunization it will elicit a response comparable to the native protein. It is therefore a good practice to assess the antigenicity of the proteins by testing their reactivity with seropositive human and animal sera which gives us a picture of the presence of factual B cell epitopes in antigen structure. Pre-screening of sera from BALB/c mice experimentally infected with *T. gondii* strain DX for 2, 3, 6, and 12 weeks demonstrated the rROP8 capacity to detect IgG class immune antibodies at each time point post-infection. However, the detection of IgM class antibodies was less effective, with antibody levels below the cutoff in three out of six individuals, 12 weeks after infection. We can compare these results with the diagnostic standard - a mixture of native *T. gondii* antigens TLA, which has been repeatedly assayed by our team on such samples from infected mice (16, 29). A comparison reveals that the detection profile of IgG class antibodies is almost identical to rROP8, while IgM class antibodies are much better detected using TLA than rROP8 protein alone. Since ROP8 protein plays a role in the formation of the parasitophorous vacuole and, following invasion, is primarily localized on PV membrane (8, 9), but despite its high production levels, it may have a detrimental impact on the generation of early IgM class antibodies. At the same time, the detection of IgM class antibodies using single antigens is generally regarded as difficult, compared to TLA (24). The phenomenon is probably related to the fact that with TLA we detect IgM class antibodies specific to many proteins at once, so the sensitivity of tests for antibodies barely detectable with a single antigen is naturally higher with TLA. On the other hand, the detection of IgM antibodies in the chronic phase of invasion presents no diagnostic value and necessitates the avidity confirmation of the IgG antibodies. The subsequent phase of the study was to evaluate the potential of the recombinant ROP8 protein as a diagnostic tool for human serodiagnosis, with a particular focus on IgG class antibody detection. Similarly, the ELISA technique was employed, and the results were analyzed using ROC analysis, to ascertain the specificity and sensitivity of the test in comparison with the TLA-based test. The recombinant rROP8 protein demonstrated a sensitivity and specificity of 100% at the cut-off value of 0.5120, a result that is fully comparable to that achieved by TLA. This is confirmed by correlation analysis in each patient group tested, which revealed a statistically significant linear relationship between TLA and rROP8 test results. It has to be noted that the TLA ROC cut-off was approximately higher by 0.2, which suggests a higher background signal of negative individuals’ serum than for rROP8 one. The utilization of the rROP8 protein could be therefore recommended, as it has the potential to minimize the likelihood of a false positive or negative result. Another team also tested the suitability of rROP8 as an antigen for toxoplasmosis diagnostics using the Western blot technique, with 100 negative and 105 positive samples giving an average sensitivity of 90% and specificity of 96%. In this case, the highest percentage of false-negative results was found in the chronically infected group (18%). In our case, using the ELISA method, with 138 negative samples and a total of 144 positive samples, we showed a specificity and sensitivity of 100% (12). The explanation for the slight discrepancies in the obtained results is the fact that the ELISA test has greater discriminatory potential and is more sensitive than the Western blotting test.

As previously stated, the selection of an appropriate, and the most immunogenic antigens for the toxoplasmosis vaccine remains a challenging aspect of vaccine development. However, an equally important aspect that is often overlooked is the cost-effectiveness and ethical aspects of testing new immunoprophylaxis solutions in an *in vivo* model. Taking into account the control groups, schedules of immunization, selection of appropriate adjuvant, and route of administration *in vivo* testing has limits resulting from actual technical feasibility. Furthermore, there is practically a limitless number of possible antigenic component combinations, e.g. in the form of epitope-based, subunit, or chimeric antigen-based formulations, and testing the antigenicity and immunogenicity of all these candidates *in vivo* would be not only financially but also ethically impossible. Thus, the first screening of antigens should be carried out *in vitro.* Due to the nature of the parasite’s life cycle, T cells play a pivotal role in fighting the infection (30), thus, it has been suggested to test the immunogenic potential of antigens by their ability to activate APC cells, such as monocytes and macrophages, *in vitro* (31, 32). This solution can provide a wealth of crucial insights. For instance, assessing the profile of the cytokines secreted by macrophages can help determine these cell’s polarity in relation to the M1 or M2 phenotype, which will then inform of the adaptive response to be activated by the evaluated recombinant antigen. However, it is possible that information regarding the production of a single cytokine will prove to be inconclusive since it does not explain the complex interactions that occur during the process of vaccination. Furthermore, the concentration of a particular cytokine that is necessary to generate an effective adaptive memory response remains unknown. This is why the recently proposed experiment involved isolation of monocytes from blood donors, transforming them into monocyte-derived DC cells, stimulating them with the rROP8, assessing the degree of activation of these dendritic cells, and then performing co-cultures of stimulated DC cells with CD4^+^ helper lymphocytes and assessing the polarity of the Th1, Th2 or Th17 response. This approach allowed a much more comprehensive analysis of innate and adaptive response cell interactions and revealed the level of APC cell activation that is sufficient for the potent activation of helper T cells.

From the initial experiments, we learned that the rROP8 antigen strongly activates the NF-kB pathway in human monocytes. This is so far the first study that assessed ROP8 antigen ability to activate this pathway which is important for the initiation of the adaptive immune response (33). Other researchers have proposed that ROP18, which is antigen from ROP2 family, just like ROP8, induces a pro-inflammatory response via the NF-κB pathway in microglia (34). In our previous study, the recombinant chimeric SAG2-GRA1-ROP1 protein achieved an *in vivo* protective efficacy of 76–86% and induced the NF-κB pathway at a similar level to that of ROP8 in the same human THP-1 monocyte *in vitro* assay (19, 35). This suggests that ROP8 may be a promising candidate for a recombinant antigen vaccine.

Further experiments presented in current study demonstrated that the rROP8 protein markedly activates mouse macrophages, resulting in elevated levels of pro-inflammatory cytokines production, including TNF-α and IL-12p40. Conversely, IL-10 production was not observed during the time frame studied after activation, which suggests an M1 type of macrophage phenotype. Macrophage activation led to the development of robust phagocytic capabilities during infection, effectively reducing the number of tachyzoites by 64% *in vitro*. The survival of *T. gondii* in macrophages may depend on TNF-α production and TNFR interaction (36). It is well documented that this parasite is capable of exploiting macrophages and DC cells in order to evade the immune system’s response (37). The detailed experimental procedure of DC cell activation and culture with Th lymphocytes provided a comprehensive explanation of the interactions that can occur when an immune system comes into contact with a ROP8 antigen. Following a 24-hour stimulation of DC cells with rROP8 antigen, an increase in the deposition of co-stimulatory maturation and activation markers, including CD80, CD83, and CD86, was observed on CD40^+^ cells (38). Twenty-four hours following stimulation, elevated levels of cytokines directly associated with DC cell activation, including TNF-α, IL-23, and IL12-p70, were observed. The greatest discrepancy between stimulated and unstimulated cells is observed in the case of TNF-α. Noteworthy, there was no increase in the expression of this cytokine at this time point in the experiment. The possible mechanism of this phenomenon is that early and strong expression resulted in high production of this cytokine, which then ceased, possibly due to notable levels of IL-10, though it also does not exhibit sustained expression. Conversely, elevated levels of IL-23 and IL-12p70 were observed, with continued expression after 24 hours. These cytokines are associated with Th17 and Th1 lymphocyte responses, respectively. It is observed that the fold change of IL-12p70 concentration is greater than that of IL-23, suggesting that it might activate Th1 response more promptly than Th17. In contrast, the production of cytokines directly related to Th lymphocytes, such as IFN-γ, IL-17A, IL-2 or IL-4 (**Supplementary Figure 3**), is not discernible. The experiments already suggest DC type 1 response; however, we investigated it further by performing a co culture experiment with T helper lymphocytes, to demonstrate their polarization. The results of the co-cultures demonstrate that the lymphocytes respond with a robust production of IFN-y, both after 48 and 96 hours of incubation, exhibiting sustained high levels of expression which results in an accumulation of this cytokine over time. A comparable effect was observed with IL-17A, although on a smaller scale with weaker expression levels. Additionally, cytokines such as IL-2 were produced, with elevated levels noted after 48 hours, followed by a subsequent decline after 96 hours. A comparable effect was observed with IL-4, which was exclusively produced by Th2 lymphocytes, and IL-10. In the case of IL-10, a significant decline was observed after 96 hours, with the expression level at 96 hours being markedly lower than that of unstimulated cells. By utilizing the control of dendritic cells alone, without lymphocytes, it was established that these cytokines are directly produced by lymphocytes and not dendritic cells. Consequently, it was also determined that these lymphocytes do not produce TNF-α in response to activation by DC cells. The results demonstrate that the stimulation had a substantial impact on DC activation, effectively converting it into a Th1/Th17-type pro-inflammatory response, which in turn resulted in the production of high levels of IFN-y. Interferon gamma is the most relevant cytokine in combating a primary *T. gondii* infection or preventing reactivation (30). This cytokine is responsible for the activation of host cells through the induction of IFN-related genes via the JAK-STAT pathway. Consequently, the concentration of this cytokine is of great importance, as *T. gondii* has the potential to inhibit STAT1 through the action of *Tg*IST. This cytokine induces several mechanisms with varying effects, which are employed to combat intracellular parasites. The efficacy of these mechanisms is different upon the specific type of *T. gondii* strain. Examples include Interferon-induced guanylate-binding protein 1 (GBP1) in human macrophages and mesenchymal stromal cells, which affect strain types I and II by disrupting the parasitophorous vacuole and destroying the infected host cell (30). Th1-type cells are the primary producers of IFN-y and IL-2, and their induction is important in combating infection. In contrast, Th2-type cells can facilitate uncontrolled parasite growth (39, 40). IL-2 production is one of the first effects of T-lymphocyte activation and it affects, among other things, the expansion of antigen-specific CD4^+^ cells, influences the differentiation of Th1 and Th2 cells, induces the proliferation of CD8^+^ memory cells and the expansion of antigen-specific clones, which is why it is so important when studying responses to vaccine antigens. In our case, the production of IL-2 induced lymphocyte proliferation, which might be why we observed such high levels of IFN-y. High levels of IFN-y may also contribute to the generation of memory macrophages through the IFN-y-related pathway (41). The role of the Th17 response in *T. gondii* infection remains not fully understood. Researchers have proposed testing this aspect of the response in vaccine trials, given that studies have indicated the involvement of these cells in vaccine-induced memory against several pathogens. The induction of Th17-type memory cells generated following vaccination results in the production of IL-17, which in turn upregulates CXCR3 ligands and recruits Th1 effector cells, leading to the production of IFN-y. It is also noteworthy that IL-17 has been demonstrated to facilitate neutrophil activation, which plays a pivotal role in the initial stages of *T. gondii* infection (42, 43). Some studies have postulated that this response may be more crucial than Th1 in the context of intracellular parasites such as *Trypanosoma cruzi*, suggesting that it may have an equally pivotal role in the control of both intracellular and extracellular parasites (44). The potential of ROP8 as a vaccine antigen in a mouse model has been demonstrated by other authors, in the context of DNA vaccines rather than recombinant protein (13, 14). These DNA vaccine studies have shown the induction of polarized Th1-type response with IFN-y production. While these results align with our *in vitro* assessment, we also propose that the Th17 response may play a pivotal role in the characteristics of the response. Overall our results of rROP8 *in vitro* immunogenicity assessment suggest that rROP8 might be a promising candidate for experimental vaccine development, which can be done as single recombinant antigen vaccine, or as a part of chimeric antigen, however a factual efficacy of an experimental vaccine is to be assessed on *in vivo* mice model in the future.

As previously stated, in addition to evaluating the immunogenic and diagnostic potential of the rROP8 protein, this study also examined this protein as a potential molecular target for new compounds with anti-*T. gondii* activity. For that purpose, we investigate the thiazolidin-4-one derivatives docking activity toward ROP8, a key protein involved in parasitophorous vacuole formation in *T. gondii*, we proposed a series of tests involving compounds exhibiting the most potent anti-*T. gondii* activity. These compounds were selected from a library of 35 new thiazolidin-4-one derivatives, which were tested in our previous study for their activity and selectivity against *T. gondii* (22). Our hypothesis was further supported by *in silico* analyses by Abdizadeh et al. which suggested that derivatives of the thiazolidin-4-one scaffold exhibit inhibitory effects on the ROP8 protein (15). Furthermore, Abdizadeh et al. expanded upon this concept by conducting a comprehensive investigation of thiazolidin-4-one derivatives using 2D/3D-QSAR modeling, molecular docking, and molecular dynamics simulations. Their findings highlighted that specific substitutions on the scaffold significantly enhance the binding affinity to ROP8, identifying key functional groups responsible for inhibitory activity. These findings align with our observations: the compounds protected rROP8 from proteolysis in the DARTS assay, indicating that they bind to ROP8, and we observed a marked decrease in the number of parasitophorous vacuoles (PV) per cell as well as a reduction in the number of dividing tachyzoites post-infection. While this suggests ROP8 engagement, it does not yet demonstrate that ROP8’s function is inhibited inside infected cells. In the previous study (22) it was shown that all active compounds in this series demonstrated superior inhibition of *T. gondii* tachyzoite proliferation compared to reference drugs *in vitro*.

In this work it was demonstrated that the compounds under investigation bind to rROP8, and unable the effective cleavage by the pronase, preventing rROP8 degradation. Noteworthy, again the compounds from the **91–95** series were generally more effective at preventing rROP8 degradation compared to derivatives from the **82–88** series. Taken together, these results give further insight into anti-*T. gondii* activity of thiazolidin-4-one derivatives and strengthen the evidence that these compounds can effectively inhibit ROP8 and consequently reduce *T. gondii* proliferation, offering a promising strategy for the development of new therapeutic agents against toxoplasmosis. Taken together, these results provide insight into the anti-*T. gondii* activity of thiazolidin-4-one derivatives and suggest that these compounds may exert their effects by engaging ROP8, which could contribute to reduced *T. gondii* proliferation. This offers a promising strategy for the development of new therapeutic agents against toxoplasmosis.

In conclusion, the results of our multidisciplinary study demonstrated the potential of the rROP8 protein for the serodiagnostics of toxoplasmosis through the detection of IgG antibodies. The results obtained in ELISA are comparable to, if not superior to, those obtained with the native proteins contained in TLA. Additionally, we proposed a protocol for the assessment of recombinant proteins immunogenic potential, which can be employed as a preliminary step prior to *in vivo* studies of experimental vaccine utility. This approach enabled us to demonstrate the high capacity of the rROP8 protein to activate human monocytes and mouse macrophages *in vitro.* To the best of our knowledge, this is the first study to utilize dendritic cells and a pure culture of helper lymphoid cells to assess the immunogenic potential of *T. gondii* proteins. Other studies have employed whole PBMC cells of humans in most cases infected with *T. gondii* at various stages of disease, to assess post antigen stimulation response. We believe that our solution offers the most comprehensive insight into the immunogenicity of the antigen, which can be used as a reliable protein preselection tool. Furthermore, our findings provide insight into a possible mechanism of anti-*T. gondii* activity for these thiazolidin-4-one derivatives: namely, their ability to bind to the ROP8 protein. These results indicate that ROP8 is involved as a potential target, suggesting that optimizing new compounds for enhanced affinity to ROP8 could potentially translate to increased antiparasitic activity.

## 5 Limitations

It is worth mentioning, that although our results support the potential usefulness of rROP8, for diagnostic, immunoprophylactic and therapeutic targeting purposes, there are certain limitations of the drawn conclusions that should be addressed in future experimental design.

Overall, the results obtained suggest that the recombinant ROP8 protein might be a promising diagnostic tool for detecting anti-*Toxoplasma* antibodies in the sera of patients infected with *T. gondii*. However, this requires further research, in particular testing a larger number of serum samples and performing tests in various laboratories to validate test sensitivity and specificity, using our estimated ELISA cut-off value.

Also, even though the rROP8 antigen is highly immunogenic in our *in vitro* assays, to confirm its actual immunoprotective capacity, as an experimental vaccine, an *in vivo* evaluation is needed to definitively demonstrate its ability to trigger a protective immune response in vulnerable host. Furthermore, although the DCs stimulation studies with rROP8 gave reliable results it is worth mentioning that applying the optimized protocol to other recombinant antigens should involve a larger number of monocyte donors.

Finally, the noticed protein interaction with tested compounds requires further research to clarify actual involvement of ROP8 antigen in observed compound-induced inhibition of *T. gondii* cyst formation in permissive cell line.

## Data Availability

The original contributions presented in the study are included in the article/**Supplementary Material**. Further inquiries can be directed to the corresponding authors.
