# Supplementary material for: Recombinant ROP8 antigen: diagnostics, immunogenicity, and therapeutic targeting in toxoplasmosis
Source: Front Immunol. 2026 Feb 5;17:1768856. doi: 10.3389/fimmu.2026.1768856 (PMC12916717; doi:10.3389/fimmu.2026.1768856)
Supplement: Supplementary file 1 [file Supplementaryfile1.docx]

Supplementary Material

# Supplementary Figures and Tables

**
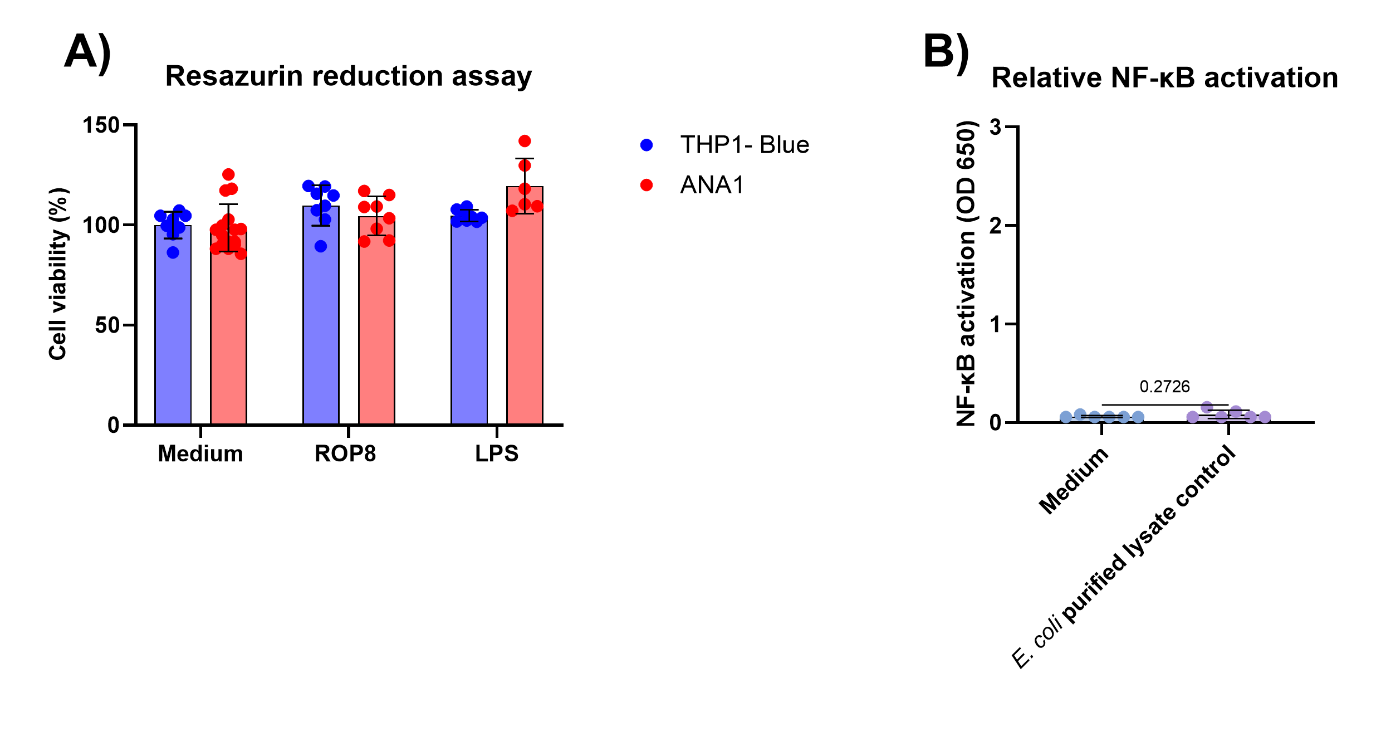
**

**sFigure 1. Supplementary data of the results of *in vitro* stimulation of APC cells. A)** Viability of THP1-Blue and ANA1 cells after 24h culture with rROP8, LPS (positive control), medium (unstimulated cells). The viability percentage was calculated using 550 nm and 600 nm OD readings. Data presented as mean and SD. **B)** Relative activation of the NF-κB pathway (OD 650 nm) after stimulation of THP1- Blue monocytes with *E. coli* Rosetta(DE3)pLysS transformed with empty pET30 Ek/LIC, induced and purified on a metal affinity column, following standard protocol for antigen purification. Lysate is diluted the same as the least concentrated antigen. Medium - unstimulated cells. Data presented as mean and SD. Data were analysed using the unpaired t-test with Welch’s correction.


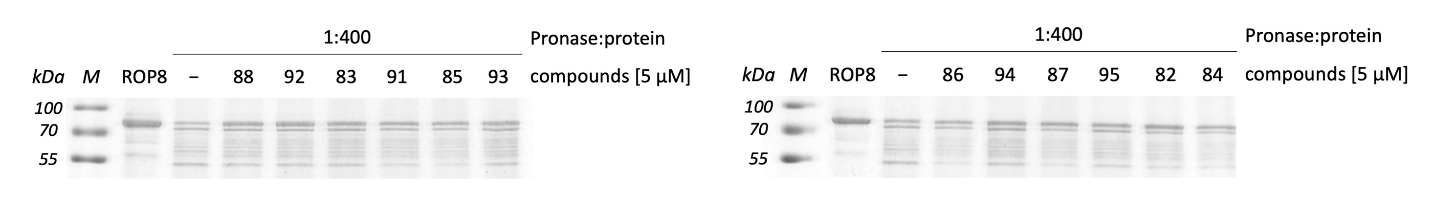


**sFigure 2. Representative SDS-PAGE gel image from the DARTS assay on the derivatives tested with the rROP8 protein.** Lane 3 (-) represents DMSO.


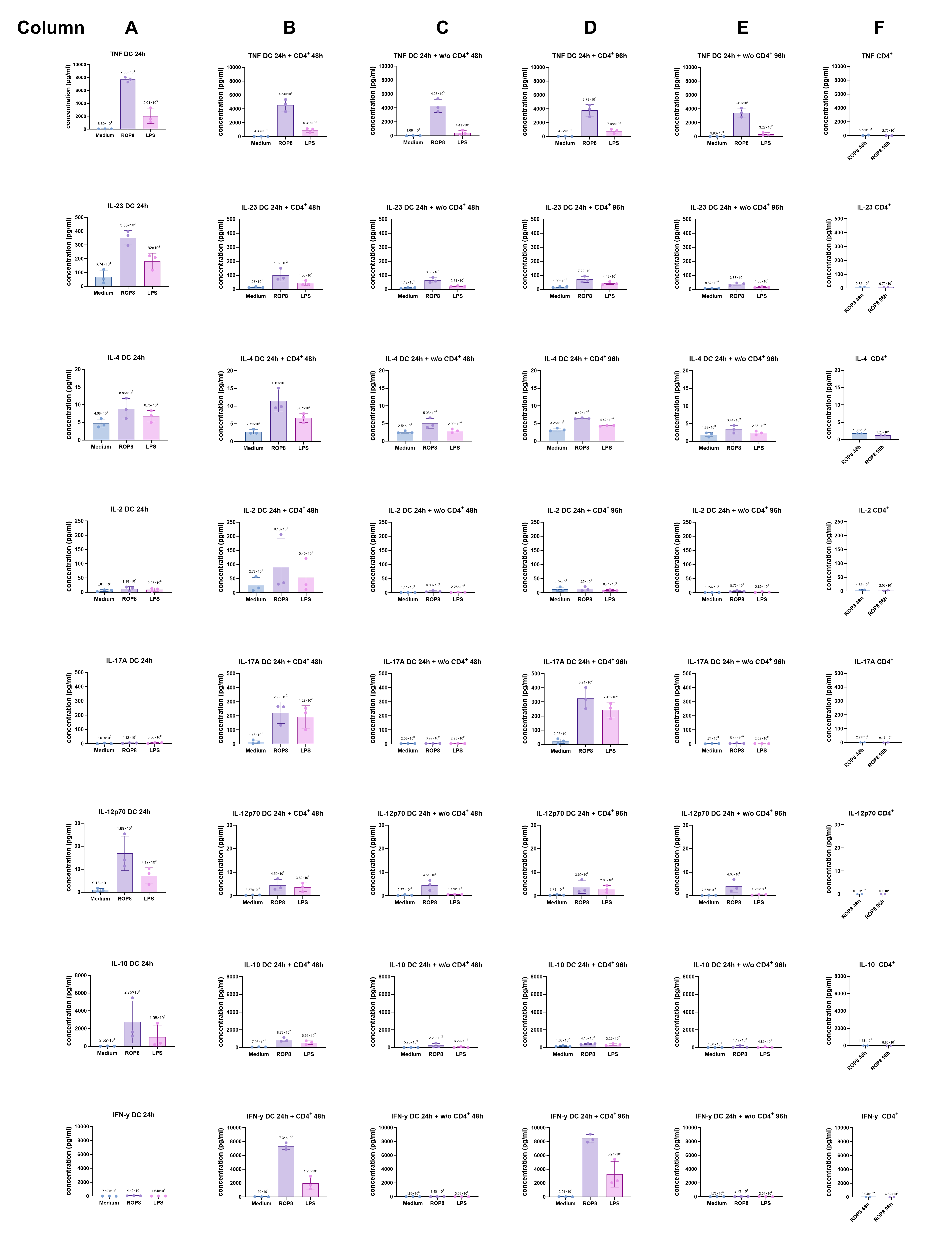


**sFigure 3. Complete results of multiplex cytokine concentrations measurements.** **Column A** represents cytokine concentration in dendritic cells (DC) culture after 24h stimulation. **Column B** represents cytokine concentration in dendritic cells (DC) culture after 24h stimulation and further 48h culture with homologous CD4^+^ T cells. **Column C** represents cytokine concentration in dendritic cells (DC) culture after 24h stimulation and further 96h culture with homologous CD4^+^ T cells. **Column D** represents cytokine concentration in dendritic cells (DC) culture after 24h stimulation and further 48h culture without homologous CD4^+^ T cells. **Column E** represents cytokine concentration in dendritic cells (DC) culture after 24h stimulation and further 96h culture without homologous CD4^+^ T cells. **Column F** represents cytokine concentration in CD4^+^ T cells culture after 48h and 96h culture with rROP8 antigen.

1 **MHHHHHHSSG LVPRGSGMKE TAAAKFERQH MDSPDRHVQQ GAGVVRPRHW**

51 **QNSEAAVSVR SPGGASPRPF HSPIEPVAFI DGEHDEDKHE GSWLEQEAAE**

101 **DVTPLPDSHT EAQTQSPSAF RRLTRRLRFW RRGVTGGSDA GEEAPQTPRP**

151 **SLRTRILQYL RRVGTFFRRD IPAAALRFFR RFRRVRQPVF PPDEFPEDVD**

201 **TNPIYFRGTD PGDVVIEELF NRIPQANVRT TSEYMQSAAD SLVSTSLWNT**

251 **GQPFRVESEL GERPRTLVRG TVLGQEDPYA YLEATDQETG ESFEVHVPYF**

301 **TERPPSNAIK QMKEEVLRLR LLRGIKNQKQ AKVHLRFIFP FDLVKDPQKK**

351 **KMIRVRLDER DMWVLSRFFL YPRMQSNLQT FGEVLLSHSS THKSLVHHAR**

401 **LQLTLQVIRL LASLHHYGLV HTYLRPVDIV LDQRGGVFLT GFEHLVRDGA**

451 **RVVSSVSRGF EPPELEARRA TISYHRDRRT LMTFSFDAWA LGLVIYWIWC**

501 **ADLPITKDAA LGGSEWIFRS CKNIPQPVRA LLEGFLRYPK EDRLLPLQAM**

551 **ETPEYEQLRT ELSAVLPLYQ** **TDGEPADLGT DDDDKSPGFS STMAISDPNS**

601 **SSVDKLAAAL EHHHHHH**

**sFigure 4.** **The amino acid sequence of recombinant ROP8 protein** (**ROP8 36-575 AA**

| SDS-PAGE | Western Blot |
| --- | --- |
| 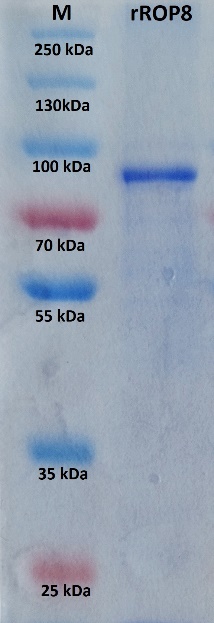 | 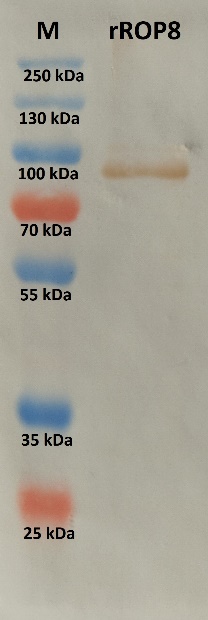 |

**sFigure 5. Representative image of a 10% SDS-PAGE gel after separation of purified rROP8 protein and western blot using HRP conjugated Anti-His tag antibodies.**

**
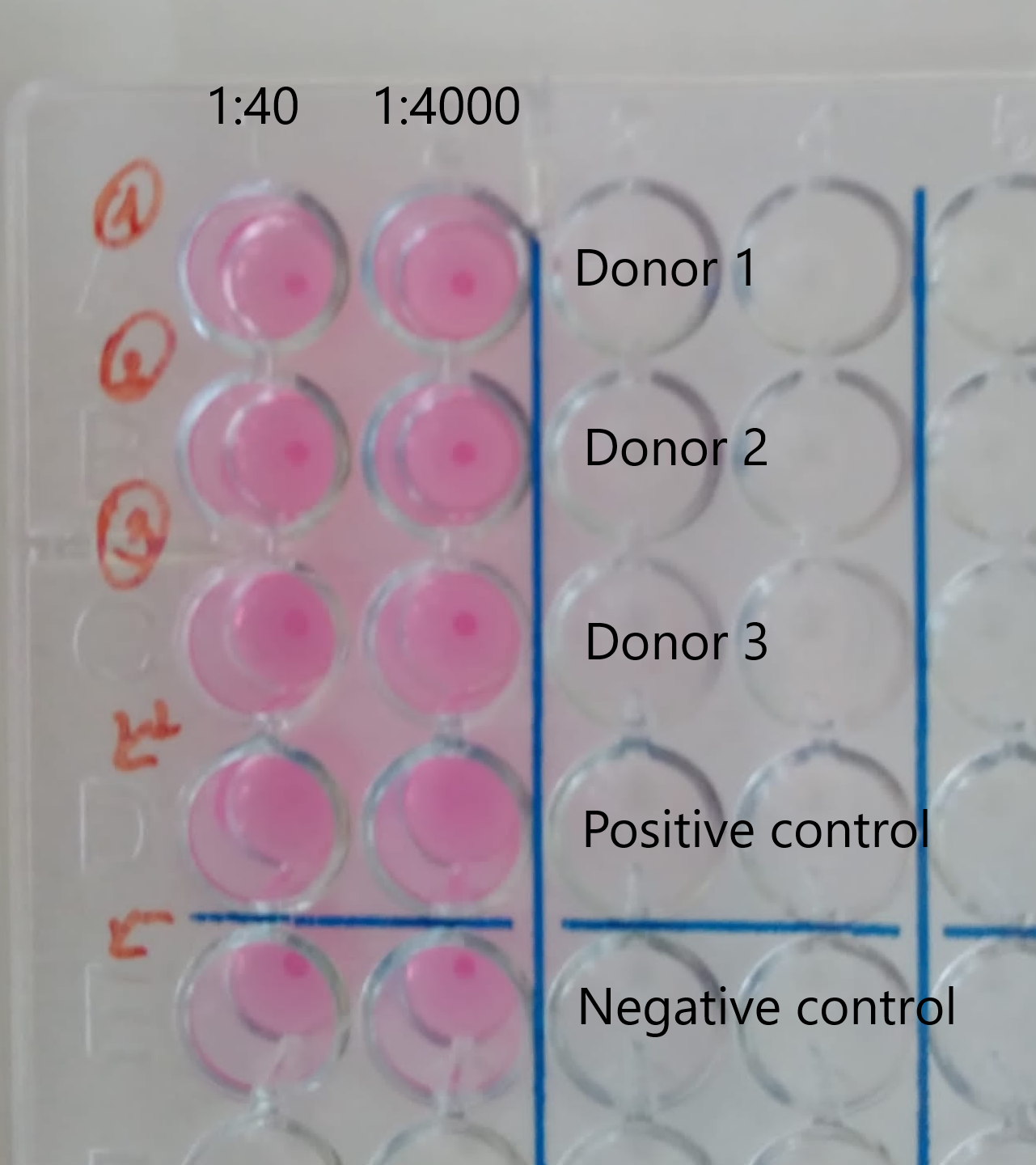
**

**sFigure 6. Donor plasma agglutination test results.** Sample dilution is marked at the top of the column.

**
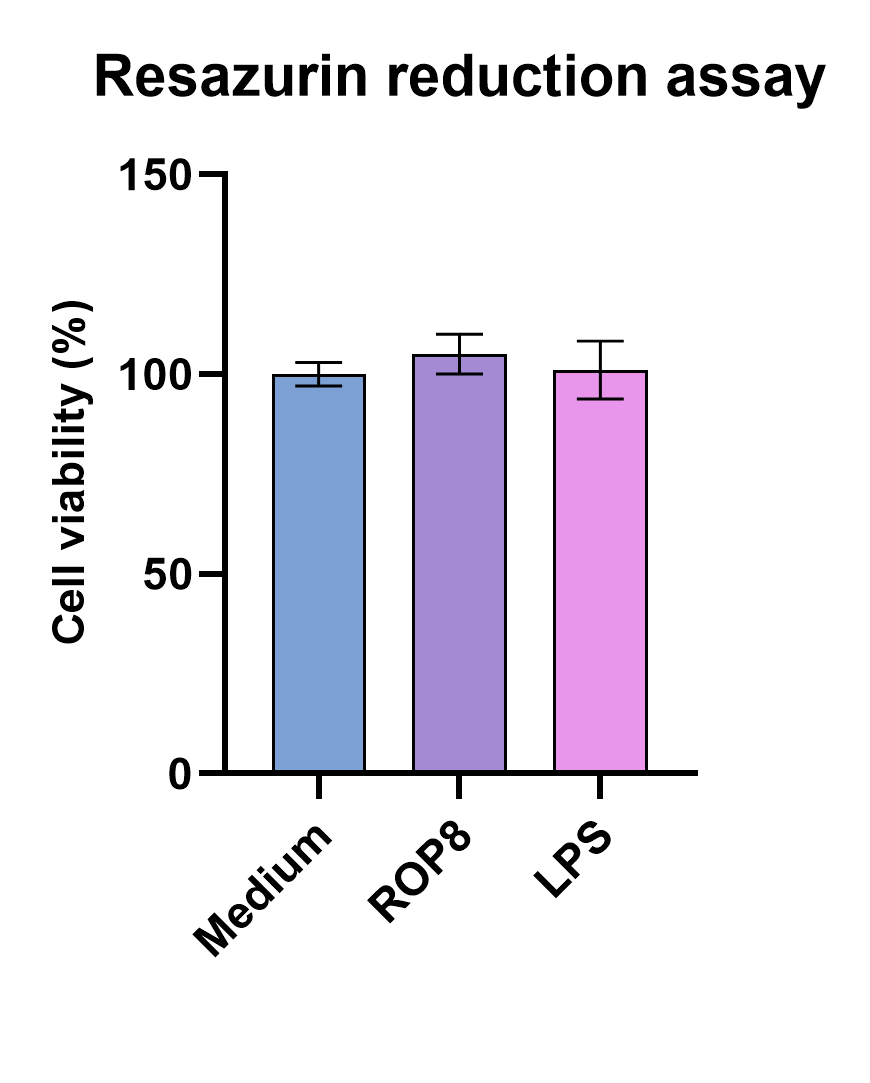
**

**sFigure 7. Viability of DC cells after 24h culture with rROP8, LPS (positive control), medium (unstimulated cells).** The viability percentage was calculated using 550 nm and 600 nm OD readings. Data presented as mean and SD.

**
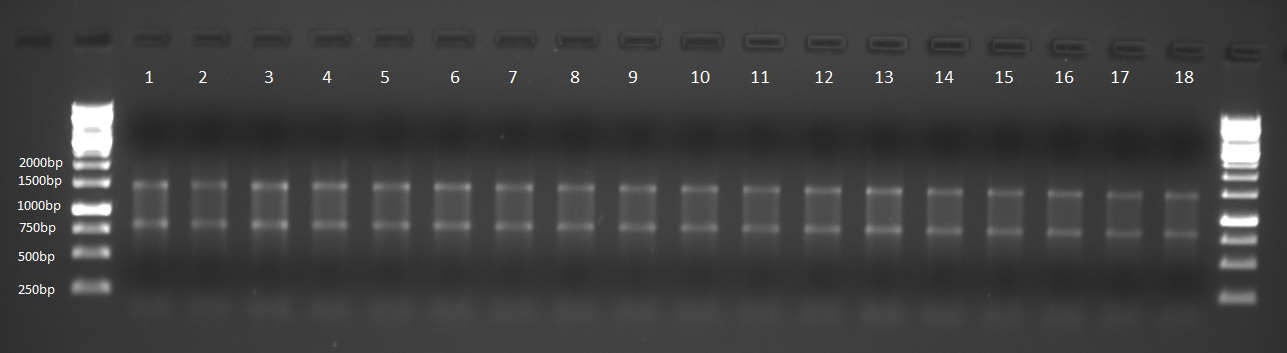
**

**Figure 8. Representative image of an RNA agarose gel electrophoresis under non-denaturing 1%/TBE conditions.** Samples were diluted in RNA gel Loading Dye (2x) (ThermoFisher) and denatured for 10 minutes at 70°C. The 18 marked RNA samples were derived from DC cells cultured for 24h after stimulation and then cultured with homologous CD4^+^ T cells for 96h. Samples came from three donors whose cells were stimulated in duplicate with rROP8, LPS or unstimulated (medium).

**sTable 1. Primer sequences used for real time PCR reactions**

| **Gene name** | **Primer sequence (from 5**′**to 3′)** | **Accession** |
| --- | --- | --- |
| IFNG | FOR: GAGTGTGGAGACCATCAAGGAAG | NM_000619 |
|  | REV: TGCTTTGCGTTGGACATTCAAGTC |  |
| IL12B (IL-12p40) | FOR: GACATTCTGCGTTCAGGTCCAG | NM_002187 |
|  | REV: CATTTTTGCGGCAGATGACCGTG |  |
| TNF alpha | FOR: CTCTTCTGCCTGCTGCACTTTG | NM_000594 |
|  | REV: ATGGGCTACAGGCTTGTCACTC |  |
| IL-10 | FOR: TCTCCGAGATGCCTTCAGCAGA | NM_000572 |
|  | REV: TCAGACAAGGCTTGGCAACCCA |  |
| IL-23A (IL-23) | FOR: GAGCCTTCTCTGCTCCCTGATA | NM_016584 |
|  | REV: GACTGAGGCTTGGAATCTGCTG |  |
| IL-2 | FOR: AGAACTCAAACCTCTGGAGGAAG | NM_000586 |
|  | REV: GCTGTCTCATCAGCATATTCACAC |  |
| IL-4 | FOR: CCGTAACAGACATCTTTGCTGCC | NM_000589 |
|  | REV: GAGTGTCCTTCTCATGGTGGCT |  |
| IL12A (IL-12p35) | FOR: TGCCTTCACCACTCCCAAAACC | NM_000882 |
|  | REV: CAATCTCTTCAGAAGTGCAAGGG |  |
| IL17A (IL-17) | FOR: CGGACTGTGATGGTCAACCTGA | NM_002190 |
|  | REV: GCACTTTGCCTCCCAGATCACA |  |
| GAPDH | FOR: GTCTCCTCTGACTTCAACAGCG | NM_002046 |
|  | REV: ACCACCCTGTTGCTGTAGCCAA |  |
| ACTB | FOR: CACCATTGGCAATGAGCGGTTC | NM_001101 |
|  | REV: AGGTCTTTGCGGATGTCCACGT |  |

**sTable 2. Mean Ct values for each donor, stimuli and gene.**

| **Sample name** | **Gene name/Ct** | | | | | | | | | | |
| --- | --- | --- | --- | --- | --- | --- | --- | --- | --- | --- | --- |
|  | **GAPDH** | **ACTB** | **TNF alpha** | **IL-23** | **IL-12p40** | **IL-12Ap35** | **IL-10** | **IFNG** | **IL-2** | **IL-17A** | **IL-4** |
| 24h DC MED. 1 | 14,59 | 10,53 | 21,49 | 28,53 | 30,97 | 24,97 | 23,81 |  |  |  |  |
| 24h DC MED. 2 | 14,68 | 10,94 | 19,92 | 27,73 | 32,01 | 25,68 | 23,71 |  |  |  |  |
| 24h DC MED. 3 | 14,69 | 10,86 | 21,00 | 28,30 | 30,85 | 25,25 | 23,45 |  |  |  |  |
| 24h DC ROP8. 1 | 15,75 | 11,85 | 22,31 | 26,34 | 24,92 | 26,26 | 25,66 |  |  |  |  |
| 24h DC ROP8. 2 | 14,72 | 12,33 | 22,01 | 24,54 | 26,99 | 25,52 | 23,56 |  |  |  |  |
| 24h DC ROP8. 3 | 16,01 | 12,51 | 21,54 | 26,21 | 25,25 | 25,51 | 25,37 |  |  |  |  |
| 24h DC LPS. 1 | 14,45 | 10,77 | 21,14 | 27,58 | 26,43 | 25,37 | 24,57 |  |  |  |  |
| 24h DC LPS. 2 | 13,90 | 11,16 | 21,54 | 26,10 | 27,41 | 25,84 | 23,66 |  |  |  |  |
| 24h DC LPS. 3 | 14,78 | 11,31 | 20,33 | 26,91 | 25,85 | 25,44 | 24,46 |  |  |  |  |
| 24h DC + CD4 48h MED. 1 | 14,80 | 11,46 |  |  |  |  | 28,02 | 29,33 | 28,54 | 31,74 | 26,72 |
| 24h DC + CD4 48h MED. 2 | 15,50 | 12,12 |  |  |  |  | 28,65 | 29,68 | 29,20 | 31,53 | 27,07 |
| 24h DC + CD4 48h MED. 3 | 15,76 | 12,15 |  |  |  |  | 28,07 | 30,25 | 29,94 | 31,39 | 27,00 |
| 24h DC + CD4 48h ROP8. 1 | 14,74 | 11,41 |  |  |  |  | 28,95 | 23,68 | 28,24 | 30,10 | 27,49 |
| 24h DC + CD4 48h ROP8. 2 | 15,49 | 12,14 |  |  |  |  | 29,29 | 23,55 | 28,28 | 29,36 | 27,39 |
| 24h DC + CD4 48h ROP8. 3 | 15,35 | 11,76 |  |  |  |  | 28,72 | 23,49 | 28,88 | 29,64 | 26,92 |
| 24h DC + CD4 48h LPS. 1 | 15,40 | 12,55 |  |  |  |  | 29,45 | 26,30 | 28,75 | 30,80 | 27,88 |
| 24h DC + CD4 48h LPS. 2 | 15,56 | 12,59 |  |  |  |  | 28,98 | 26,29 | 28,94 | 30,22 | 27,42 |
| 24h DC + CD4 48h LPS. 3 | 15,62 | 12,34 |  |  |  |  | 28,60 | 25,27 | 29,30 | 29,96 | 26,96 |
| 24h DC + CD4 96h MED. 1 | 14,25 | 11,72 |  |  |  |  | 26,98 | 29,02 | 28,89 | 31,66 | 26,46 |
| 24h DC + CD4 96h MED. 2 | 14,58 | 11,29 |  |  |  |  | 26,38 | 28,70 | 28,39 | 31,61 | 25,80 |
| 24h DC + CD4 96h MED. 3 | 14,34 | 11,17 |  |  |  |  | 26,06 | 28,34 | 27,42 | 31,22 | 25,49 |
| 24h DC + CD4 96h ROP8. 1 | 13,78 | 11,43 |  |  |  |  | 28,47 | 25,42 | 28,41 | 30,72 | 26,68 |
| 24h DC + CD4 96h ROP8. 2 | 14,67 | 11,45 |  |  |  |  | 28,26 | 25,49 | 29,99 | 31,07 | 26,94 |
| 24h DC + CD4 96h ROP8. 3 | 14,68 | 11,24 |  |  |  |  | 27,54 | 23,69 | 28,46 | 30,08 | 26,01 |
| 24h DC + CD4 96h LPS. 1 | 14,45 | 11,43 |  |  |  |  | 27,88 | 26,81 | 28,98 | 31,19 | 26,81 |
| 24h DC + CD4 96h LPS. 2 | 14,69 | 11,80 |  |  |  |  | 27,52 | 27,60 | 29,10 | 31,25 | 26,62 |
| 24h DC + CD4 96h LPS. 3 | 15,61 | 12,14 |  |  |  |  | 27,53 | 25,76 | 30,02 | 30,99 | 26,40 |
